# Supplementary material for: Interference haptic stimulation and consistent quantitative tactility in transparent electrotactile screen with pressure-sensitive transistors
Source: Nat Commun. 2024 Aug 21;15:7147. doi: 10.1038/s41467-024-51593-2 (PMC11339070; doi:10.1038/s41467-024-51593-2)
Supplement: Supplementary file 1 — Supplementary Information [file 41467_2024_51593_MOESM1_ESM.pdf]

Interference haptic stimulation and consistent quantitative tactility in transparent electrotactile screen with pressure-sensitive transistors

Kyeonghee Lim[1,2,†], Jakyoung Lee[1,2,†], Sumin Kim[1,2,†], Myoungjae Oh[1,2,†], Chin Su Koh[3], Hunkyu Seo[1,2], Yeon-Mi Hong[1,2], Won Gi Chung[1,2], Jiuk Jang[1,2], Jung Ah Lim[4,5,6], Hyun Ho Jung[3*], Jang-Ung Park[1,2,3,4,7*]

[1]Department of Materials Science and Engineering, Yonsei University, Seoul 03722, Republic of Korea

[2]Center for Nanomedicine, Institute for Basic Science (IBS), Yonsei University, Seoul, 03722, Republic of Korea

[3]Department of Neurosurgery, Yonsei University College of Medicine, Seoul 03722, Republic of Korea

[4]Yonsei-KIST Convergence Research Institute, Seoul, 03722, Republic of Korea

[5]Soft Hybrid Materials Center, Korea Institute of Science and Technology (KIST), Seoul 02792, Republic of Korea

[6]Division of Nanoscience and Technology, KIST School, University of Science and Technology (UST), Seoul, 02792, Republic of Korea

[7]Graduate Program of Nano Biomedical Engineering (NanoBME), Advanced Science Institute, Yonsei University, Seoul, 03722, Republic of Korea

[*]Corresponding authors. E-mail address: jang-ung@yonsei.ac.kr (J.-U.P.); junghh@yuhs.ac (H.H.J)

† These authors contributed equally to this work.

This PDF file includes:

Supplementary Notes 1 to 12

Supplementary Figures 1 to 47

Supplementary Tables 1 to 4

Supplementary Videos 1 to 3

Supplementary References 1 to 48

Supplementary Notes

Supplementary Note 1. Principle of electro-tactile system

The human somatosensory system contains a variety of sensory neurons that detect different types of sensations. Among these, on the surface of the skin, tactile sensory neurons called mechanoreceptors are responsible for distinguishing and feeling touch. Mechanoreceptors emit receptor potentials in response to external tactile stimuli such as vibration, indentation, or stretching, and these signals are transmitted to the brain for touch perception. Four types of mechanoreceptors have been identified based on their threshold or adaptation properties to external stimulation, each exhibiting a distinct firing pattern and electrical frequency of stimulation (Supplementary Fig. 1). For instance, the mechanoreceptor located in the Merkel's disk is accountable for skin indentation and fine texture discrimination, with its stimulation frequency of 0.4 – 10 Hz (Supplementary Table 1). Application of pulsed waveform electrical stimulation to the skin allows the current to penetrate the epidermis and stimulate these mechanoreceptors. By adjusting the frequency or current density, diverse electrical stimuli can be generated, resulting in varied tactile sensations. Leveraging these principles, an electro-tactile system can realize tactile sensations through electrical stimulation without the necessity of a mechanically vibrating actuator[1–4].

Supplementary Note 2. Mechanical fatigue tests of the TPIEA device

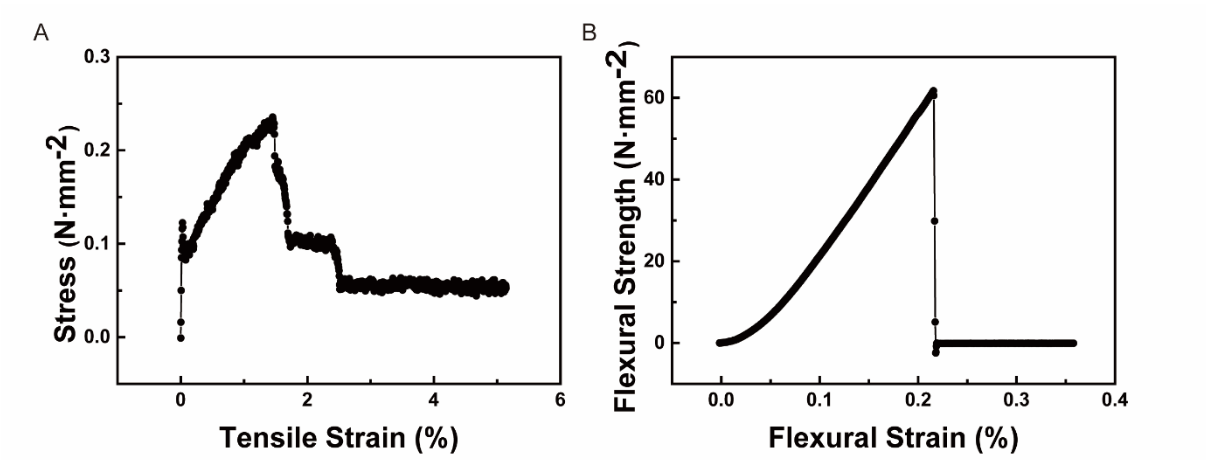

Supplementary Note Fig. 1. Mechanical properties of TPIEA device. **(A)** Stress-Strain curve and **(B)** flexural Stress-Strain curve of TPIEA device.

The mechanical characteristics of TPIEA are assessed across three primary classifications, namely tensile strength, flexural strength, and impact resistance. First, tensile strength test using a Universal Testing Machine (Instron 3367) with a 30,000 N load cell at 5 mm min^{-1} and a 50 mm grip distance determined the tensile strength as 0.24 MPa, with a yield strain of 1.4815%, indicative of a brittle specimen. Post-yield, constant stress was observed due to the elastomeric PDMS. Second, flexural strength test using a Universal Testing Machine (Instron 34SC-5) with a 5,000 N load cell at 0.7 mm min^{-1} and a 25 mm support distance determined the flexural strength as 61.7 MPa. Fracture occurred at a bend angle of 1.213° with a loading pin displacement of 0.8192 mm, indicative of a brittle material. The flexural strength and bend angle were significantly influenced by the glass substrate, similar to the tensile strength results. Lastly, impact resistance test, conforming to Izod impact test criteria, was conducted using a Digital Impact Tester (QM700A, Qmesys) with an IH1 pendulum. The test, which measures the energy required to break a standardized specimen, showed that applying 3.7 kJ m^{-2} resulted in the complete breakage of the device.

Supplementary Note 3. The safety of TPIEA's electrical stimulation on skin and tactile nerve

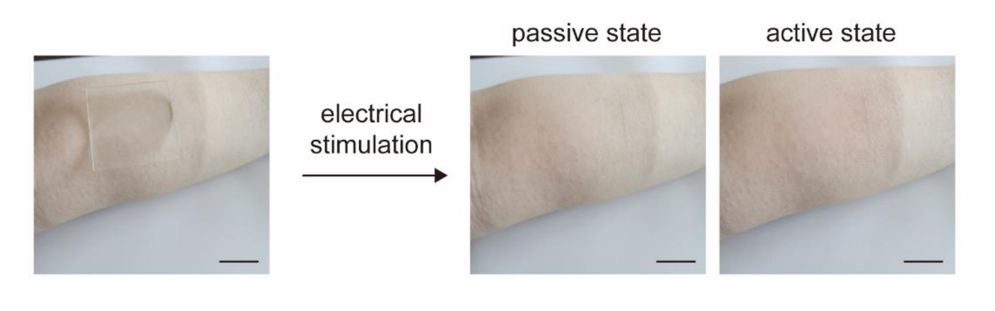

Supplementary Note Fig. 2. Safety of TPIEA on human skin. Photographs of skin condition before stimulation and skin condition without (passive state) and with electrical stimulation (active state) after 1 hour. Scale bars, 2 cm.

Initially, we compared the effects of applying electrical stimulation for one hour with no stimulation on the skin of the arm using the TPIEA device. The stimulation parameters included a frequency of 10 Hz, pulse width of 10 ms, and intensity of 0.015 A mm^{-2}. Results indicated the absence of skin irritation or redness upon removal of the device in both the control and stimulation states, as illustrated in Supplementary Note Fig. 2.

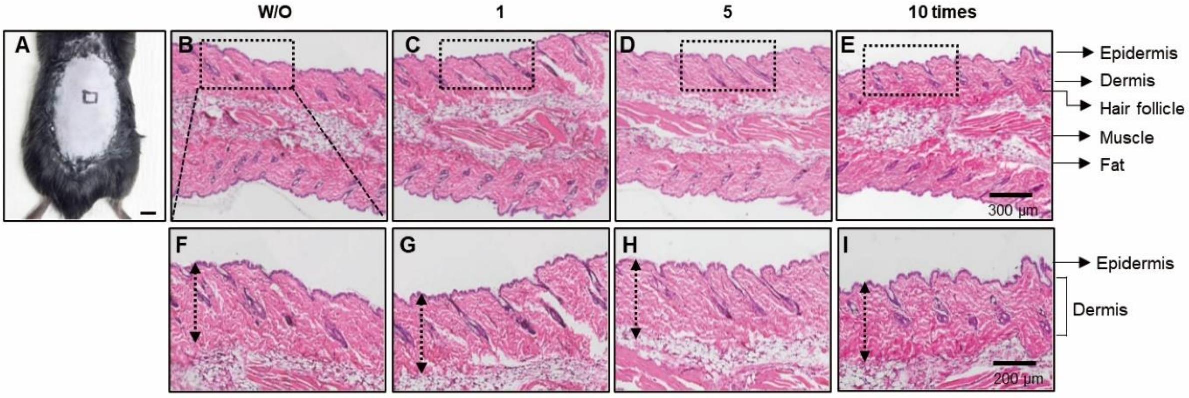

Supplementary Note Fig. 3. H&E staining showing the histological structure of the mouse dorsal skin. The structure of the epidermis in the **(A)** dorsal skin of mice. Scale bar = 1 cm. **(B, F)** control; wo **(C, G)** 1 times **(D, H)** 5 times and **(E, I)** 10 times after 0.015 A mm^{-2} stimulation.

Subsequently, following the application of stimulation on the back of mouse using the TPIEA device, epidermal samples were collected, fixed in paraffin, and subjected to hematoxylin and eosin (H&E) staining to assess changes in dermis-epidermis thickness and the presence of burn marks. Stimulation was administered for 6 minutes, comprising 5 minutes of electrical stimulation and 1 minute of rest, with experiments conducted under varying durations (no stimulation, 1 set - 6 minutes, 5 sets - 30 minutes, and 10 sets - 60 minutes). The stimulation parameters remained consistent at 10 Hz frequency, 10 ms pulse width, and 0.015 A mm^{-2}. The stimulation parameters remained consistent at 10 Hz frequency, 10 ms pulse width, and 0.015 A mm^{-2}. As shown in the Supplementary Note Fig. 3, H&E staining revealed the architecture of epidermis, dermis, hair follicles and muscles of each group did not under the extent of 0.015 A mm^{-2} stimulation (compare double arrow of Supplementary Note Fig. 3F and 3I).

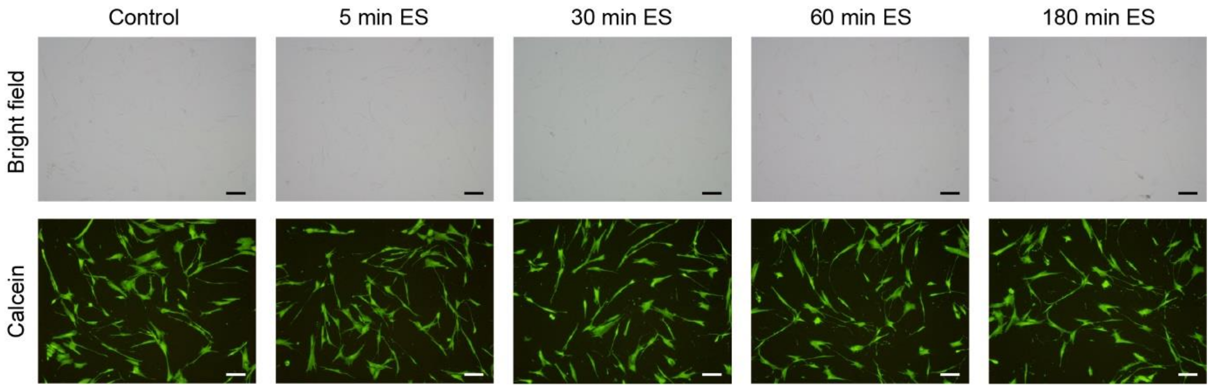

Supplementary Note Fig. 4. Calcein AM assays of SH-SY5Y cells cultured in media containing the tactile devices for 0 minutes, 5 minutes, 30 minutes, 60 minutes, and 180 minutes of electrical stimulation (ES). All scale bars = 100 μm.

Third, we tested cell viability at different time points when electrical stimulation was applied using the Calcein AM assay. We cultured SH-SY5Y cells, which are a human neuroblastoma cell line, on the device and applied electrical stimulation with a current intensity of 0.015 A mm^{-2}, a frequency of 10 Hz, and a pulse width of 10 ms for 0 minute (control group), 5 minutes, 30 minutes, 60 minutes, and 180 minutes. As a result of Calcein AM, fluorescence images confirmed with an optical microscope exhibit consistent trends for each group (Supplementary Note Fig. 4). After applying electrical stimulation for 180 minutes, only a few cells died, and overall cell viability was maintained.

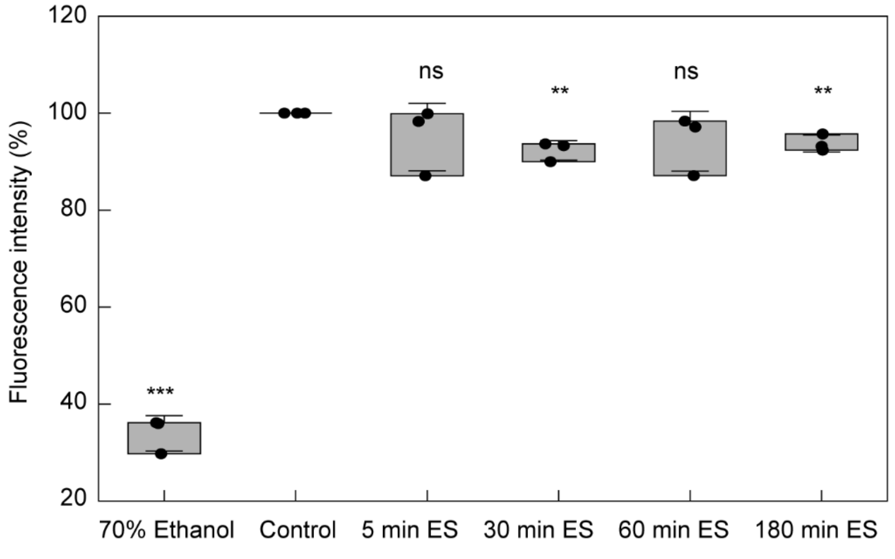

Supplementary Note Fig. 5. Fluorescence intensity of Calcein AM based on the control group (0 minute) (n=3). To compare cell death levels, a positive control group treated with 70% ethanol was included. Significant differences were analyzed with the unpaired student's t-test

and marked as ns ($p > 0.05$), ** ($p <0.01$) and *** ($p <0.001$). The exact p values are, from left to right, 6.218×10^{-6}, 2.75×10^{-3}, and 3.425×10^{-3}.

In addition, for quantitative analysis, fluorescence intensity was calculated from the calcein-acetoxymethyl results. To assess cell death, 70% ethanol was used as a positive control group. Supplementary Note Fig. 5 shows the results for each group, with the viability of the ethanol-treated group (positive control), 0 minute (control), 30 minutes, 60 minutes, and 180 minutes of electrical stimulation as 33.96 ± 2.98%, 100%, 95.09 ± 5.69%, 92.32 ± 1.65%, and 94.21 ± 5.04%, and 93.74 ± 1.43%, respectively. The experiments were performed with a sample size of 3 for each assay. The fluorescence intensity remained above 93% after electrical stimulation, regardless of the duration.

The safety of TPIEA's electrical stimulation was validated through these three experiments, indicating the absence of adverse impacts on human skin or tactile nerves following prolonged exposure to electrical stimulation.

Supplementary Note 4. Assessing compliance with the safety regulations to TPIEA

The safety of TPIEA has been demonstrated through three key factors: (1) assessment of the current level employed, (2) adherence to established general standard criteria, and (3) evaluation of the leakage current level.

(1) Assessment of the current level employed.

To demonstrate that nerve paralysis does not occur for amount and duration of current applied in this work, evaluation of the current density used in the experiment was conducted. The maximum current applied was calculated through multiplying maximum current density applied to skin with the area of the electrode, which is expressed as $Current\ density \times Area\ of\ the\ electrode\ (A)$, where: $A = \pi \times (250\ \mu m)^2 \approx 0.2\ mm^2$. The maximum current applied was calculated as 15 mA. According to the design guidelines of device for developing safe electrical current delivery system to the human body[5], electrotactile system presented in this work is a haptic technique that corresponds to perception among the methods of electrical application. Moreover, according to the international standard[6], the current density level in this work, which is applied for 10 ms time duration, corresponds precisely to the AC-2 Perceptible phase, which is the level of current that does not cause muscular contraction or irreversible clinical effects.

(2) Adherence to established general standard criteria.

To verify the compliance of the electrical stimulation presented in this work with international standards, IEC 60601-2-10, which is equivalent to GB-9706 standards for medical devices, was referred[7]. IEC 60601-2-10 outlines safety requirements for nerve stimulation, specifying that for pulse outputs, the maximum energy per pulse should not exceed 300 mJ with a 500 Ω load resistance, and the maximum output voltage should not exceed 500 V in open circuit conditions.

The maximum energy per pulse was calculated using the formula $E = I^2Rt$. With a maximum current density of 0.075 A mm^{-2} applied to a circular electrode of 250 μm radius (0.2 mm^2 area), the maximum current was determined to be 0.015 A (15 mA). For a 500 Ω load resistance, the maximum power was 0.1125 W, resulting in pulse energies of 1.125 mJ and 56.25 mJ, both well within the 300 mJ limit, confirming compliance with IEC 60601-2-10 standards.

To verify compliance with the IEC 60601-2-10 standard's second criterion, which limits the maximum output voltage to 500 V under open circuit conditions, the maximum voltage applied during device operation was calculated using the Ohm's law ($V = IR$). The maximum current density applied was 0.075 A mm^{-2}, and the electrode area was approximately 0.2 mm^2, resulting in a maximum current of 0.015 A (15 mA). The total resistance expressed as series circuit ($R_{Device} + R_{skin}$), combining the device impedance (555 Ω) and dry skin resistance (10 kΩ), was calculated as 10,555 Ω. Using the Ohm's law ($V = IR$), the maximum output voltage was calculated as $0.015\ A \times 10555\ \Omega = 158.325\ V$. This value is well below the 500 V limit, confirming the safety of the electrical stimulation utilized in this work.

In conclusion, conducted verification process confirmed that the electrical stimulation used in this study complies with the IEC 60601-2-10 safety requirements for medical electrical equipment, proving the safety of the TPIEA. The maximum energy per pulse was 1.125 mJ and 56.25 mJ, well below the 300 mJ limit. Additionally, the maximum output voltage under open circuit conditions was 158.325 V, significantly lower than the 500 V threshold. These results demonstrate that electrical stimulation presented in this work meet international safety standards.

In addition to IEC 60479-1, other international standards for a low-frequency electrotherapy equipment, namely Japanese Industrial Standards (JIS), JIS T 2003:2005[8] and JIS C 9335-2-209:2007[9], are also satisfied, which are stated as:

- The current must be lower than 20 mA (with 1 kΩ resistance).
- The frequency must be lower than 1,200 Hz.
- The voltage must be lower than 200 V (with 1 kΩ resistance)
- The pulse energy must be lower than 120 mJ (with 1 kΩ resistance).

Regarding the safety and reliability of the device, all cognitive experiments were performed under the supervision of a neurosurgeon, which is reflected in the manuscript.

(3) Evaluation of the leakage current level.

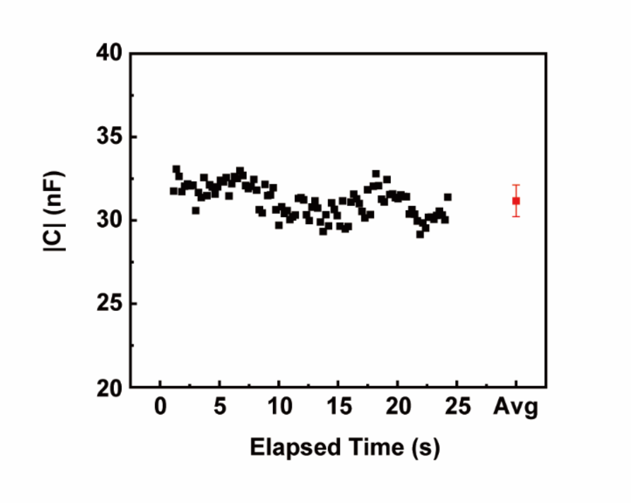

Supplementary Note Fig. 6. Capacitance of TPIEA device.

Furthermore, additional computations were performed to demonstrate that even in the event of voltage leakage, the applied current remains at a safe level. Voltage leakage can potentially result in personal injury if excessive current flows through the human body due to

such leakage. The problematic levels of leakage current commence at 10 mA, posing a risk of medical injury[10,11]. The formula for leakage current, $I = 377VC$, was employed to determine the current leakage that would arise in the event of device malfunction. As explained in Comment 3, the total maximum operating voltage calculated from the current density is assumed to be 150 V. With a measured capacitance of 31.17 nF for the device, the maximum leakage current was calculated to be 0.00176436 A, corresponding to 1.7 mA (Supplementary Note Fig. 6). Based on this analysis, it has been established that the experiment was conducted within a safe current threshold, as the potential leakage current within the experiment remains below 2 mA.

Supplementary Note 5. Somatosensory evoked potential analysis

The SEP can provide a quantitative measure to evaluate how various types of electrotactile stimuli applied to the fingertips are perceived[12–14]. The stimulation sites of SEPs are divided into the median nerve and tibialis nerve in peripheral nerves, and recordings can be possible in the cortex and spinal cord. Meaningful changes in the latency and potential difference of peaks occur depending on the location of the stimulation and recording sites. The SEP peaks are identified by the combination of the polarity (positive and negative) of the peak, represented by the alphabet P and N, and the nominal post-stimulus delay (in milliseconds) at which the peak occurs[15] (Supplementary Fig. 8). The latency, post-stimulus peak time, and peak potential difference of peaks are used as indicators in SEP analysis. SEP peaks can be divided into short-latency SEP, measured within 50 ms, and long-latency SEP, measured after 50 ms. Short-latency SEP signifies the initial neurophysiological response in the somatosensory pathway to the stimulus. Among the peaks, N20 represents the earliest cortical potential recorded in the cortex when the median nerve is stimulated and is widely used in clinical prognostic questions due to its accuracy and stability compared to later cortical waveforms (such as P45, N60, and P/N100). Therefore, when analyzing the SEPs perceived in the cortex by stimulating electrotactile sensation on the fingertips, N20 is used as the criteria, measured in pair with P23[16].

Supplementary Note 6. Pressure sensing for electrotactile actuator

The thickness (d_1) of the air-dielectric at this low pressure is a little thinner than 50 µm, the initial thickness of the air-dielectric. Therefore, the drain current becomes slightly increased, and the pressure is measured at a small level. Simultaneously, the finger is weakly stimulated by the current from the electrotactile actuator, which is directly in contact with the skin of the finger, because of the small contact area. On the contrary, when high pressure is applied to the TPIEA, the thickness (d_2) of the air-dielectric is shorter than d_1, and the relative change in drain current has a higher value than that with the thickness of d_1. In addition, an increase in the contact area allows the subjects to perceive the electrotactile sensation strongly.

Supplementary Note 7. Electro-thermal actuation

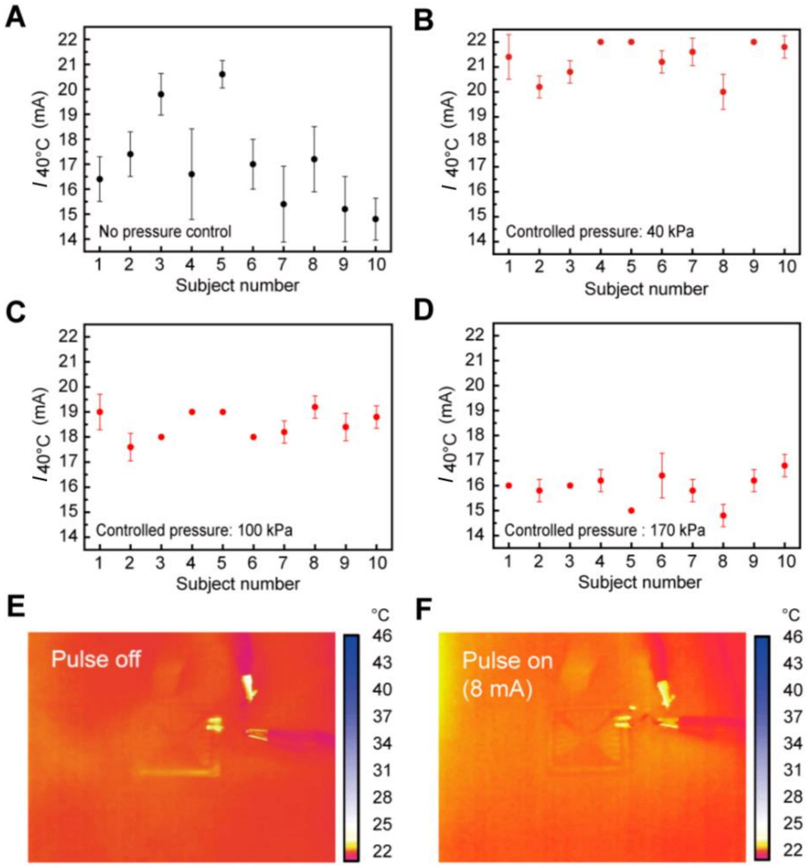

Supplementary Note Fig. 7. Thermal actuation test using electro-tactile actuator.

Current amplitudes where 10 subjects felt the warmth which was similar to the heat that can be felt from objects with a temperature of 40°C with **(A)** uncontrolled pressure, **(B)** 40 kPa, **(C)** 100 kPa, and **(D)** 170 kPa of controlled pressure. **(E)** Observation of exothermic reaction through an infrared camera before and **(F)** after applying the pulse stimulation of electro-tactile actuation.

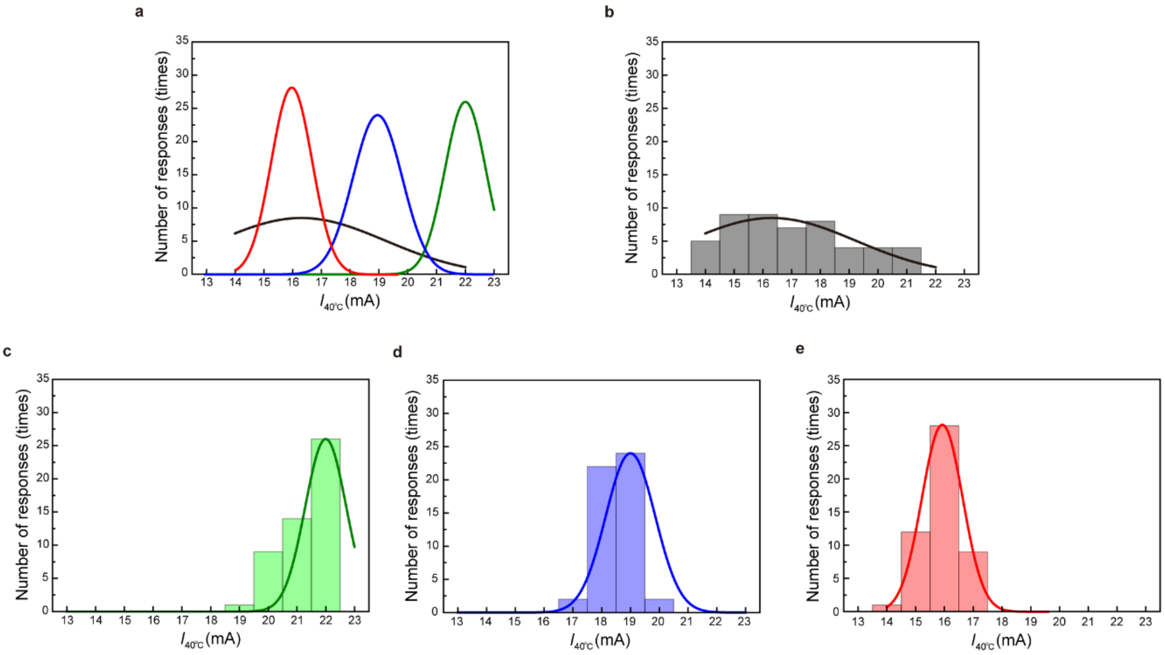

Supplementary Note Fig. 8. Distribution of the response to the thermal actuation.

(A) Comparison of Gaussian distribution for four conditions of pressure control. **(B,C,D,E)** Statistical distribution of the response for 10 subjects to the thermal actuation at the uncontrolled pressure (B), at the controlled pressure of 40 kPa (C), 100 kPa (D), and 170 kPa (E).

Behind the epidermis of human skin, there are various mechanoreceptors activated by mechanical forces including touch mechanoreceptors. The touch mechanoreceptor is classified as the low-threshold mechanoreceptor which is more sensitive to weak stimulation. On the other hand, a nociceptor is activated by mechanical stimulation with a high-threshold level and is classified as a high-threshold mechanoreceptor. It elicits a painful sensation to protect the body from noxious irritants. In addition to mechanoreceptors, there are other types of somatosensory neurons that detect different stimuli other than mechanical force, such as temperature and chemical substances. For instance, thermoreceptors fire the receptor potential

when it senses heat or cold, and most of them are also nociceptive neurons that cause the painful sensation as a response to the intense heat or cold. It is still unknown how the thermoreceptor detects all ranges of temperature, but the process of responding to temperature above a certain value has been clarified based on the activation of ion channels[3]. Transient receptor potential vanilloid 1 (TRPV1) is a representative ion channel involved in the mechanism in which human perceives the sense of warmth. TRPV1 is activated by the temperature above 43°C, allowing the passage of calcium ion (Ca^{2+}), sodium ion (Na^{+}), and potassium ion (K^{+}) from the inside to the outside of thermoreceptor[17]. It creates the potential difference across the membrane of thermoreceptor, which elicits the receptor potential. When the receptor potential is transmitted to the central nervous system, the human can perceive the sense of warmth.

Ten healthy adult subjects (age, 28-40 years; five males and five females) participated in the electro-thermal perception test. Just before the test, subjects cleaned their finger using a pure towel. We increased the stimulation current gradually started from 11 mA, and the stimulation was stopped when the subject response to feel the heat. Once the subject felt the heat, they were told to find the temperature similar to the actuated artificial heat. The subjects said the artificial heat was close to the temperature of 40°C. The current level that made the subject feel the heat ($I_{40°C}$) was recorded, and the average of $I_{40°C}$ was calculated. Calculated value was used to plot the distribution of $I_{40°C}$. This process was repeated four times for following condition (1) No pressure control, (2) the pressure was controlled at 40 kPa, (3) the pressure was controlled at 100 kPa, and (4) the pressure was controlled at 170 kPa. Also, the test was repeated five times for each subject.

Through an experiment in the previous section, we found the possibility for the TPIEA to give subjects the sense of warmth at a high amplitude of stimulating current. Based on this experiment, we designed a further study for electro-thermal actuation. 10 subjects were asked

to report their sensation rendered on their fingertips. The sensation was implemented through the electrical stimulation. The TPIEA applied the current to the electrodes while increasing the amplitude of the current starting from 14 mV with a step size of 1 mV. The frequency and pulse width of the current were 10 Hz and 10 ms, respectively. As a result, the subjects felt warmth, which was similar to the heat that can be felt from the objects with a temperature of 40°C, at the current amplitude of 14 mA or above. The results are plotted in Supplementary Note Fig. 7A, which shows the current level at which the subjects felt the artificial heat ($I_{40°C}$). $I_{40°C}$ was distributed in various ranges from 14 mA to 21 mA, and the responses of the test which was repeated 5 times per subject were inconsistent when the finger pressure was not controlled. Therefore, we examined the thermal perception in three conditions of pressure control (40 kPa, 100 kPa, and 170 kPa) in the same way as electro-tactile stimulation of tactile perception test. When the finger pressures were controlled at 40 kPa, the response range was relatively narrow, from 20 mA to 22 mA, and the average standard deviation (SD) was considerably decreased to 0.17 whereas it was 0.41 without controlling finger pressure (Supplementary Note Fig. 7B and Supplementary Note Fig. 8). Similarly, $I_{40°C}$ for the condition of pressure controlled at 100 kPa and 170 kPa were locally distributed, and the average values of SD were also low, 0.13 for controlled pressure of 100 kPa and 0.14 for 170 kPa, showing a significant difference with the SD before controlling the finger pressure (Supplementary Note Figs. 7C and D). Consequently, the higher the finger pressure applied, the lower $I_{40°C}$ was measured.

Furthermore, to ensure that the device did not emit actual heat, we observed the TPIEA using an infrared thermal camera and compared the surface temperature before and after the application of electrical stimulation (Supplementary Note Figs. 7E and F). As a result, the temperature was always lower than 22°C even though the 8 mA of electrical pulse was

generated by the device, and accordingly there was no heat of 40°C. It was verified that TPIEA can also function as an electro-thermal actuator, allowing people to experience heat on the display of electronic devices without exothermic reaction.

Supplementary Note 8. A quantifiable strategy for distinguishing between touch and pain sensation in an electrotactile system

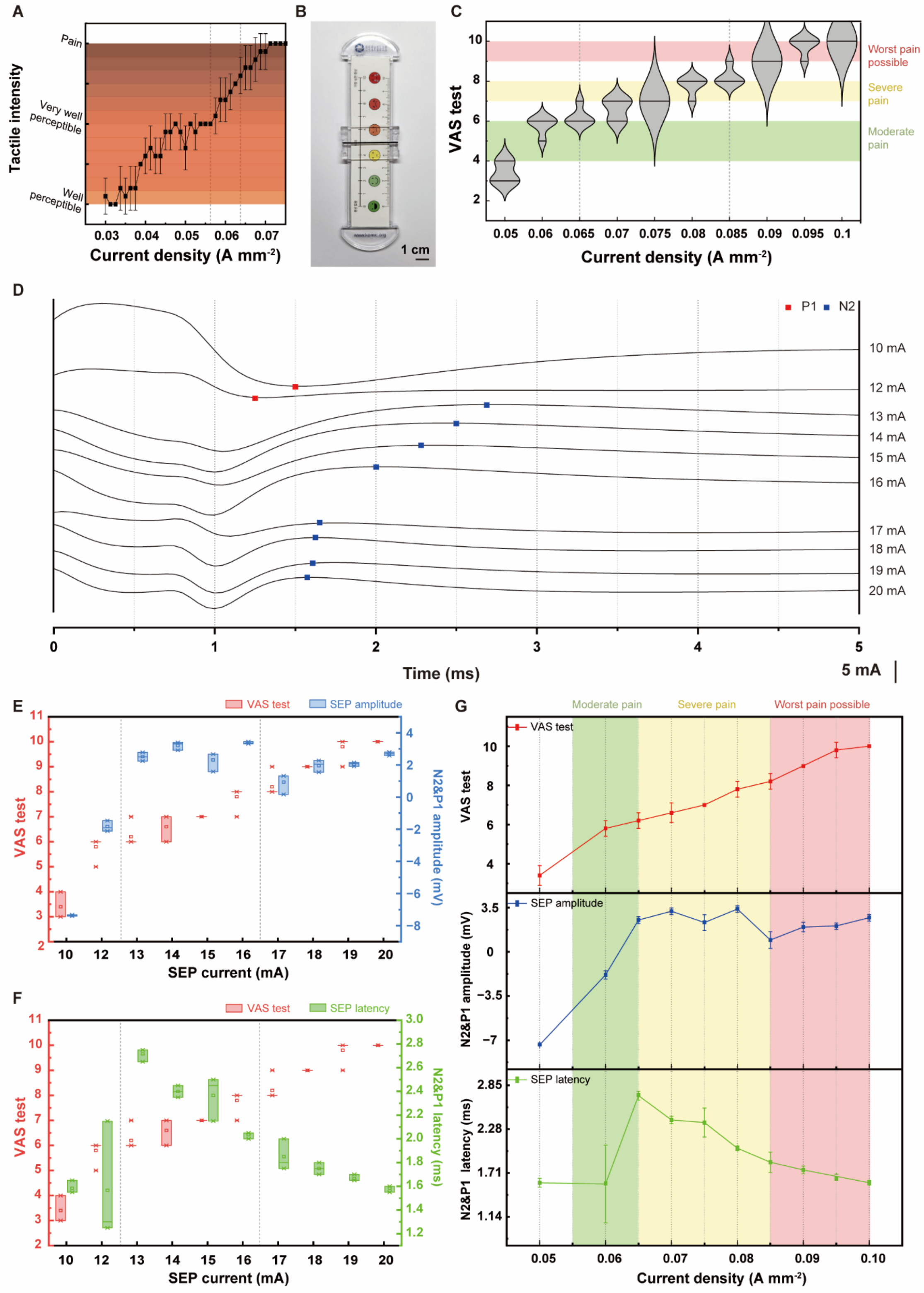

Supplementary Note Fig. 9. Experiments for distinguishing and analyzing the differentiation between pain and touch sensation. **(A)** Tactile intensity according to current density from 0.03 A mm^{-2} to 0.075 A mm^{-2} with a spacing of 0.0125 A mm^{-2}. **(B)** Tool used for pain assessment in the Visual Analog Scale (VAS) test. Scale bar = 1 cm **(C)** VAS test results from 0.05 A mm^{-2} to 0.1 A mm^{-2} **(D)** SEPs ranging from 0 ms to 5 ms with amplitudes of 10, 12, 13, 14, 15, 16, 17, 18, 19, and 20 mA (Average count for one signal = 300, Frequency = 4.7 Hz). **(E)** Comparison of SEP amplitude of N2 and P1 with VAS results according to SEP current changes **(F)** Comparison of SEP latency of N2 and P1 with VAS results according to SEP current changes **(G)** Graph showing the differentiation of pain level based on changes in VAS results, SEP amplitude, and SEP latency when current density changes.

Additional experiments were conducted to effectively distinguish between touch and pain sensations and to control unpleasant electrotactile sensations. Initially, in order to ascertain the precise threshold for the sensation of “Pain” in electrotactile perception, we conducted a reassessment of the intensity of electrotactile sensation ranging from the point of being "Well perceptible" to that of "Pain" by reducing the current density interval. The stimulation parameters employed were a frequency of 10 Hz and a pulse width of 10 ms. As a result, the threshold current density marking the differentiation between the “Pain” and “Very well perceptible” levels is 0.0575 A mm^{-2}, with the onset of the “Pain” level being detected at 0.0638 A mm^{-2} (Supplementary Note Fig. 9A).

In order to establish a clear distinction between pain and touch sensation and to develop a quantifiable strategy, Visual Analog Scale (VAS) test was conducted. The VAS test is a method for assessing pain in humans, enabling the representation of pain intensity through

a numerical score on a pain scale. The VAS test classifies pain levels within the ranges of 4-6 as “Moderate pain”, 7-8 as “Severe pain”, and 9-10 as “Worst pain possible,” a system endorsed by the World Health Organization for indicating pain severity in its guidelines for analgesic use[18]. Based on the current density collected in the previous experiment, a VAS test was performed with stimuli ranging from 0.05 A mm^{-2} to 0.1 A mm^{-2}. In this test, “Moderate pain” indicates a level of irritation but not significant discomfort (Supplementary Note Fig. 9B).

A VAS test was administered alongside an electrotactile sensation intensity assessment to five subjects (age, 25-30 years; three males and two females) with the finger pressure calibrated to medium (100 kPa). Following the establishment of a VAS rate of 5 for the electrotactile sensation corresponding to “Very well perceptible” level, we recorded the responses when the current density was increased from 0.05 A mm^{-2} to 0.1 A mm^{-2}. As a result, at a peak current density of 0.075 A mm^{-2}, which had been the highest level in the preceding experiment, the VAS test yielded an average response of 7 denoting “Severe pain,” while at 0.1 A mm^{-2}, the response escalated to 10, indicating “Worst pain possible” (Supplementary Note Fig. 9C). The experiment facilitated the representation of pain levels on a scale, but since the VAS test also relies on the subjective assessment of the subjects, we re-measured the SEPs that preceded it to establish a baseline of electrotactile sensation in order to collect accurate quantifiable data.

SEP data was first measured at 10 mA, where no pain was felt, and then SEP data was collected and compared from 12 mA to 20 mA, where the very well perceptible phase began with three subjects (age, 25-30 years; two males and one female). The experimental conditions and environment are the same as in experiment 2, except for the average count in Method. The average count used for a single collection was upscaled to 300 for tighter results. The indicators utilized to correlate pain and touch sensation with SEP were the emergence, latency, and

amplitude of P1-2 and N1-2 corresponding to the initial peak[19–21]. The results showed that P1 was observed at 10 mA and 12 mA around 1-2 ms, while N2 was observed from 13 mA onwards. The latency of P1 at 10 mA and 12 mA remained relatively stable at 1.583 ± 0.057 ms and 1.567 ± 0.505 ms, respectively, although the amplitude exhibited an decrease from 7372 ± 42.14 μV to 1828 ± 333.16 μV. Moreover, within the range of 13 mA to 16 mA, the latency of N2 decreased from 2.717 ± 0.058 ms to 2.033 ± 0.029 ms, accompanied by an increase in amplitude from 2511 ± 270.022 μV to 3209 ± 259.356 μV. Conversely, for the 17 mA to 20 mA range, the latency of N1 notably decreased from 1.85 ± 0.13 ms to 1.583 ± 0.029 ms, and the amplitude tended to increase gradually from 933 ± 666.27 μV, which was lowered once, to 1957 ± 371.15 μV, 2048 ± 121.20 μV, and 2693 ± 108.01 μV (Supplementary Note Fig. 9D).

Following the integration of the quantitative analysis of SEP with the outcomes of the VAS test, the analysis revealed that the pain sensation could be categorized into three segments based on the presence or absence of N2: sensation below 13 mA, sensation below 16 mA with no discernible amplitude trend, and sensation up to 20 mA with a pattern of amplitude fluctuation (Supplementary Note Fig. 9E-F). By aligning the SEP data with the current density and electrotactile sensation data obtained from the prior VAS test, a clear differentiation between tactile sensation and pain was achievable. Ranging from 0.055 A mm^{-2} to 0.065 A mm^{-2} was identified as “Moderate pain”, 0.065 A mm^{-2} to 0.085 A mm^{-2} as “Severe pain”, and 0.085 A mm^{-2} to 0.1 A mm^{-2} as “Worst pain possible” (Supplementary Note Fig. 9G).

In conclusion, the differentiation between pain and touch sensation based on current density was successfully achieved. Based on the VAS and SEP analysis, it was determined that the pain sensation at a current density of 0.075 A mm^{-2}, which corresponds to the existing "Pain" level, was at the "Severe pain" level, which is the low level of actual pain. Also, given that touch perception within “Perceptible” to “Well perceptible” level is adequate for a realistic

tactile simulation, a current density of 0.05 A mm^{-2} or lower was utilized in all applications to ensure subjects were exposed only to non-painful stimulation.

Supplementary Note 9. Electrotactile sensation in different humidity conditions

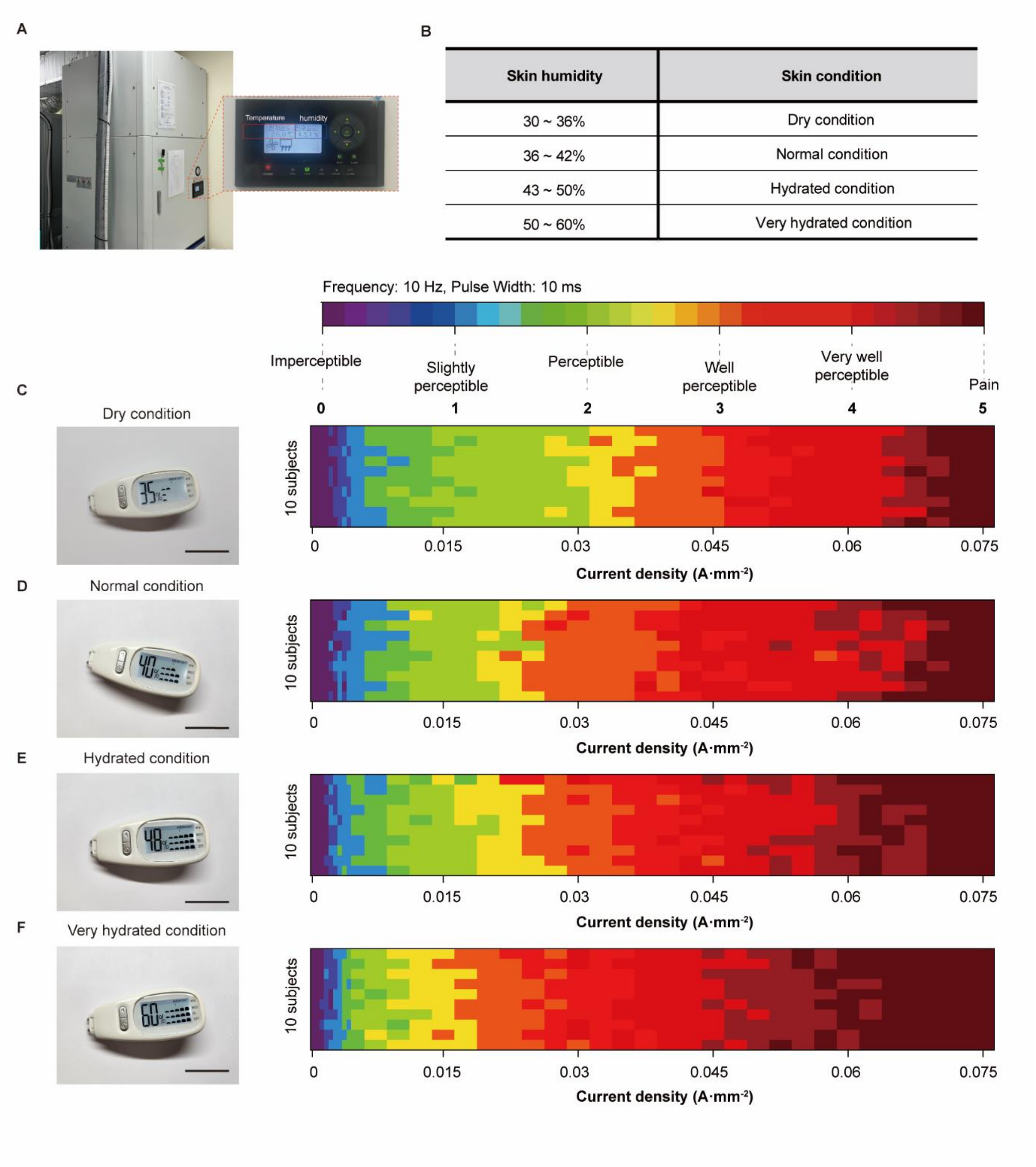

Supplementary Note Fig. 10. Experiment on electrotactile sensation in a varying humidity setting. **(A)** Photograph of a temperature and humidity control unit. A temperature and humidity control unit was utilized to maintain a consistent level of humidity for controlled environment: 26°C and under 43.1% humidity. **(B)** Table categorizing skin conditions according to skin

humidity. Hygrometer readings and corresponding electrotactile intensity for each skin condition on **(C)** dry condition, **(D)** normal condition, **(E)** hydrated condition, and **(F)** very hydrated condition. All scale bars = 3 cm.

Skin humidity is a significant factor affecting the electrotactile sensation, as it can impact skin hydration and conductivity. Specifically, skin moisture levels can enhance skin hydration and conductivity, consequently altering the conductivity of the stratum corneum layer[22–25]. A decrease in humidity leads to an increase in skin-electrode impedance, while an increase in humidity results in the opposite effect. Given that skin-electrode impedance is influenced by both pressure and skin humidity, we also focused on measuring the variations in electrical sensation corresponding to different humidity levels.

The laboratory maintained temperature and humidity levels using a temperature and humidity control unit (Supplementary Note Fig. 10A). The temperature and humidity control unit in our experiment operates on the principle of regulating both temperature and humidity within a closed environment to predetermined levels. This is achieved through a combination of sensors, cooling/heating elements, and a humidifier/dehumidifier system. The sensors continuously monitor the environmental conditions, and the control system adjusts the heating or cooling elements to maintain the desired temperature. Similarly, the humidifier adds moisture to the air when the humidity drops below the set level, while the dehumidifier removes excess moisture when the humidity exceeds the desired level. Humidity regulation within the device was achieved by integrating and encapsulating the system in a vacuum environment with an air-gap.

Human skin condition was categorized into dry (30-36%), normal (36-42%), hydrated (43-50%), and very hydrated (50-60%) based on the humidity levels (Supplementary Note Fig. 10B)[26]. Prior to the experiments, subjects measured the humidity levels of their skin by skin analyzer (RoHS compliant; China) and then proceeded electrotactile intensity experiment following the same method as in Experiment 3, with finger pressure initially set to a standardized medium pressure of 100 kPa. It indicated that an increase in skin moisture levels led to a heightened electrotactile perception at lower current densities. The threshold current density required to reach the "Perceptible" level showed no significant variation across dry, normal, and hydrated skin conditions at 0.0125A mm^{-2}. However, with higher current densities, the thresholds for reaching the "Very well perceptible" and "Pain" levels gradually decreased. Notably, in the highly hydrated condition, all stages, including the "Perceptible" level, exhibited lower thresholds compared to the normal condition, with the pain stage threshold being the lowest at 0.0575 A mm^{-2} (Supplementary Note Fig. 10C-F). This investigation aimed to explore the impact of skin humidity on electrotactile sensation, with results confirming that skin humidity can influence the perception of electrotactile stimulation. It is important to note that all experiments, except for this study, focused on normal skin conditions with a maintained humidity level of 38-42%. Additionally, conditions involving cognitive experiments, such as handling electronic devices with wet hands (humidity exceeding 50%), were not considered.

Supplementary Note 10. Two-point discrimination test

This TPD test is a commonly utilized method in clinical settings to gauge tactile spatial acuity, and determines the minimum distance between two stimulation points that can be identified as separate on the skin, known as the TPD threshold[27]. When the skin is stimulated at two points separated by a distance below the TPD threshold, the individual points are perceived as a single point. For instance, the TPD threshold for the human forearm is 30 mm, while the fingertip exhibits a TPD threshold of 2 mm on average (the threshold range within ~ 5 mm due to individual variations), contingent on the density distribution of mechanoreceptors[28,29]. Precise implementation of electrotactile sensation is essential because of this fingertip's ability to discern high-resolution sensations compared to other body parts.

Supplementary Note 11. Interference stimulation

Interference occurs when two electric fields intersect, resulting in interference waves of varying amplitudes and frequencies. Two sets of electrodes are necessary to produce a single interference wave, with the frequency of the generated interference wave being the offset frequency of the two waves, and the maximum amplitude being the sum of the amplitudes of the two waves. The optimal stimulation location for interference is where the sum of the vectors of the two electric fields is the greatest, indicating stimulation at the center of the body rather than directly over the electrodes.

Interference stimulation operates based on two assumptions: the interference phenomenon caused by the superposition of the low-pass filtering property and the electric field. First, the low-pass filtering property is a theory that the neural membrane does not

consciously respond to electrical fields above 1 kHz[30]. Next, let's call the altering current generated by a pair of anode and cathode as I_1, and the altering current generated by another pair of anode and cathode as I_2. Due to the interference phenomenon where the electric fields created by these two currents overlap, a new electric field can appear[31,32]. Simply put, when the frequency of I_1, f_1, is above 1 kHz with an amplitude of Z, and the frequency of I_2, f_2, is $f_2 = f_1 + \Delta f$ ($\Delta f \leq 200$ Hz) with an amplitude of Z, due to the superposition of the two electric fields, an offset frequency of Δf is generated. Theoretically, an altering modulation electric field with a maximum amplitude of $2Z$ is created when it is perfect interference condition. This is based on the formation of beating (acoustic) through superposition when two overlapping electric fields have different frequencies, resulting in the creation of a wave in the form of an envelope. The generated wave is referred to as an envelope wave.

The electric field generated by the interference phenomenon caused by the superposition of I_1 and I_2 approached mathematically is expressed as follows.

a_1 = wave 1

a_2 = wave 2

a = envelop wave

t = time

ω_n = frequency of wave n

ω = frequency of envelop wave

A_n = amplitude of wave n

A = amplitude of envelop wave

A_{max} = maximum amplitude of envelop wave

A_{min} = minimum amplitude of envelop wave

μ = modulation index value

E_{AM} = envelope modulation amplitude

$A_1 \sin \omega_1 t = a_1$ ... (1)

$A_2 \sin \omega_2 t = a_2$ ... (2)

$A \sin \omega t = a$ ... (3)

To calculate A, start with A_2 as the reference and add the amplitude change of Wave 1.

$A = A_2 + a_1$ ... (4)

$A = A_2 + A_1 \sin \omega_1 t$ ... (5)

Accordingly, A_{max} is obtained when $\sin \omega_1 t$ is +1, and A_{min} is obtained when $\sin \omega_1 t$ is –1.

$A_{max} = |A_1 + A_2|$ ... (6)

$A_{min} = |A_1 - A_2|$ ... (7)

Add equation 6 and equation 7.

$A_{max} + A_{min} = 2A_2$

$A_2 = (A_{max} + A_{min}) / 2$ ... (8)

Subtract equation 7 from equation 6

$A_{max} - A_{min} = 2A_1$

$A_1 = (A_{max} - A_{min}) / 2$ ... (9)

$A_1/A_2 = (A_{max} - A_{min}) / (A_{max} + A_{min}) = \mu$ ... (10)

When perfect modulation occurs, μ has a value of 1.
Also, according to the principle of beating, ω is as follows.
$\omega = \omega_1 - \omega_2$ (beating frequency) = offset frequency... (11)

Therefore, equation 3 can be summarized as follows.
$a = A_2(1 + \mu \sin \omega_1 t) \sin (\omega_1 - \omega_2)t$ ... (12)

The ideal value of μ is 1. However, due to factors such as the attenuation of amplitude during the propagation of the electric field through the medium and the phase discrepancies among waves, μ typically assumes a value of 1 or less. The difference between the maximum (A_{max}) and minimum (A_{min}) amplitudes during this process is termed the envelope modulation amplitude (E_{AM}), which signifies the strength of the actual stimulation within the envelope wave. E_{AM} varies based on the magnitude of μ, increasing with higher values of μ.

To achieve a high μ, it is necessary for the amplitudes A_1 and A_2 at the point of interference to be similar. E_{AM} is maximized at the location where the values of A_1 and A_2 interfere most similarly, leading to stimulation, while at other locations, the E_{AM} does not have enough values to surpassing the threshold, preventing stimulation.
Therefore, Interference stimulation can be described as a stimulation method using the phenomenon of interference, and to summarize:

1. Stimulation occurs at the position where the μ is maximized. When A_1 and A_2 are the same, the center position is the position where μ is maximized. By adjusting the position of μ, stimulation can be carried out at different locations.

2. The intensity of the stimulation is E_{AM}, so theoretically it can deliver a stronger stimulus than A_1 and A_2.

3. The frequency of stimulation is the offset frequency, which is the difference between the two waves.

Supplementary Note 12. VR and AR application with TPIEA

Virtual reality (VR) and augmented reality (AR) technologies are advancing rapidly across multiple sectors including education, entertainment, and healthcare, offering immersive experiences. While these technologies predominantly rely on visual and auditory stimuli, the provision of tactile feedback has been somewhat limited, often utilizing vibrations from devices like gloves[33]. In contrast, electrotactile technology could present a promising solution by delivering a sense of touch through high-resolution and diverse stimulation methods, thereby enhancing the tactile experience in VR environments[34,35]. An illustrative application involves the integration of electrodes into VR gloves or attaching them to the back of the hand to elicit tactile sensations via electrical stimulation when interacting with virtual objects. Furthermore, the incorporation of electrotactile technology with AR devices facilitates interactions between virtual elements and the physical world. The amalgamation of VR, AR tools, or full-body suits with electrotactile technology holds potential for diverse applications in areas such as education, gaming, medical training, and remote robot manipulation[36]. To leverage these opportunities effectively, critical considerations include precise control of stimulation, development of personalized feedback systems, implementation of wireless and portable solutions, as well as the commercialization and standardization of these technologies.

Supplementary Figures

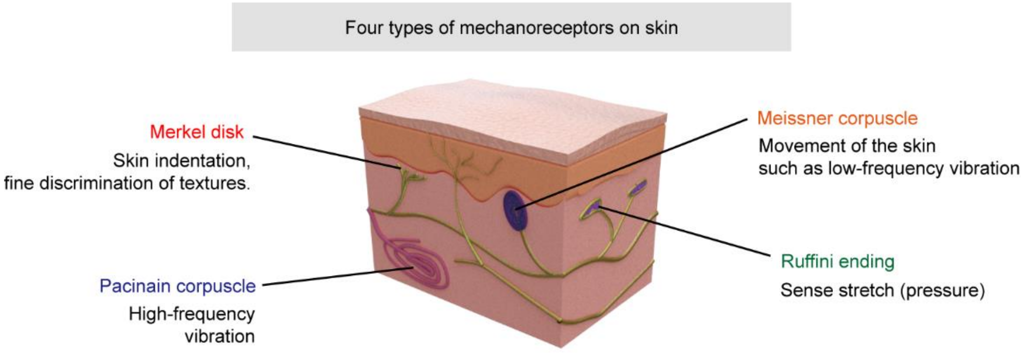

Supplementary Fig. 1. Four types of mechanoreceptors on skin. Schematic image showing the four types of mechanoreceptors, the sensory neurons responsible for tactile perception.

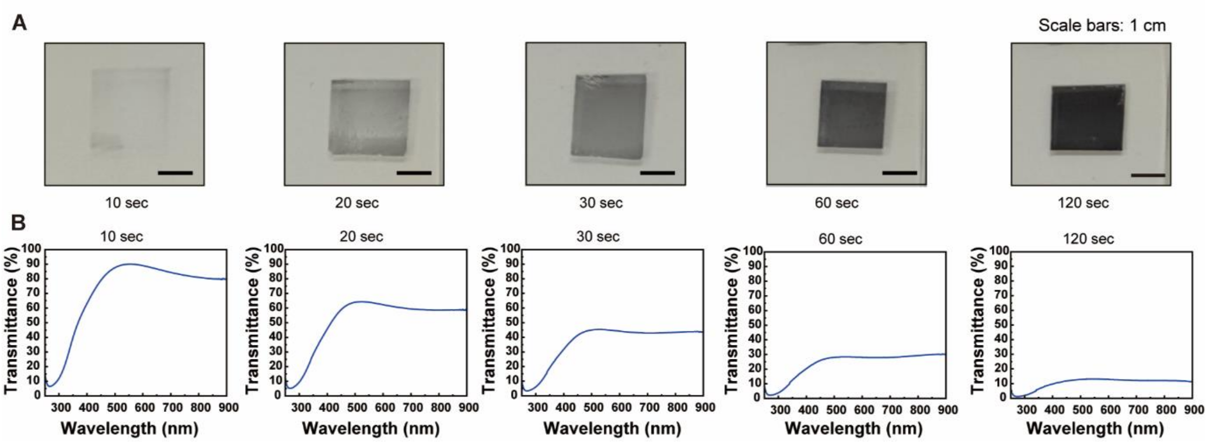

Supplementary Fig. 2. Transmittance variation with electroplating time.

(A) Photographs of Pt nanocluster coatings on ITO varying with electroplating time from 10 s to 120 s. **(B)** Graphs of transmittance measured with corresponding coating times. When the wavelength corresponding to visual light is 550 nm, the transmittance was 89.7% at 10 s, 63% at 20 s, 47% at 30 s, 29% at 60 s, and 12% at 120 s.

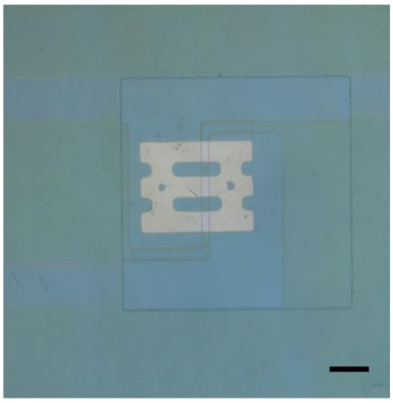

Supplementary Fig. 3. Optical image of the field-effect transistor of pressure sensor. Scale bar, 100 μm.

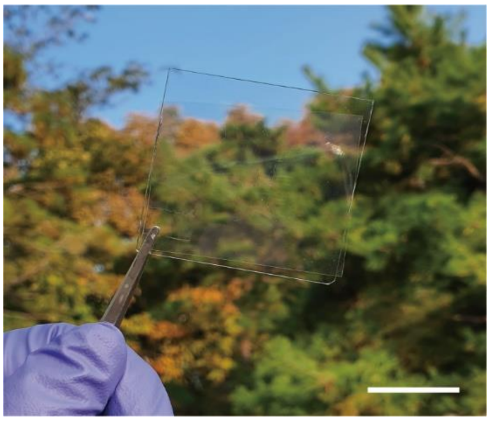

Supplementary Fig. 4. Photograph of the transparent electrotactile actuator with pressure sensor. Scale bar, 2 cm.

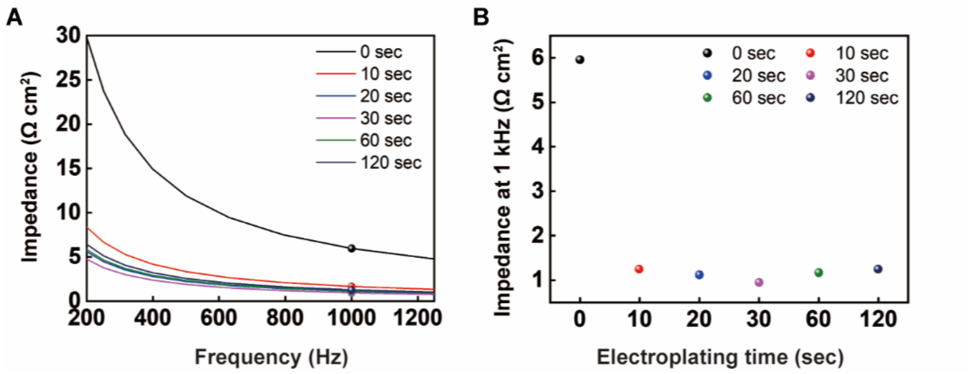

Supplementary Fig. 5. Impedance value of ITO electrode as a function of Pt nanostructure electroplating time.

(A) Variation of impedance value with frequency range. **(B)** Impedance value at 1000 Hz.

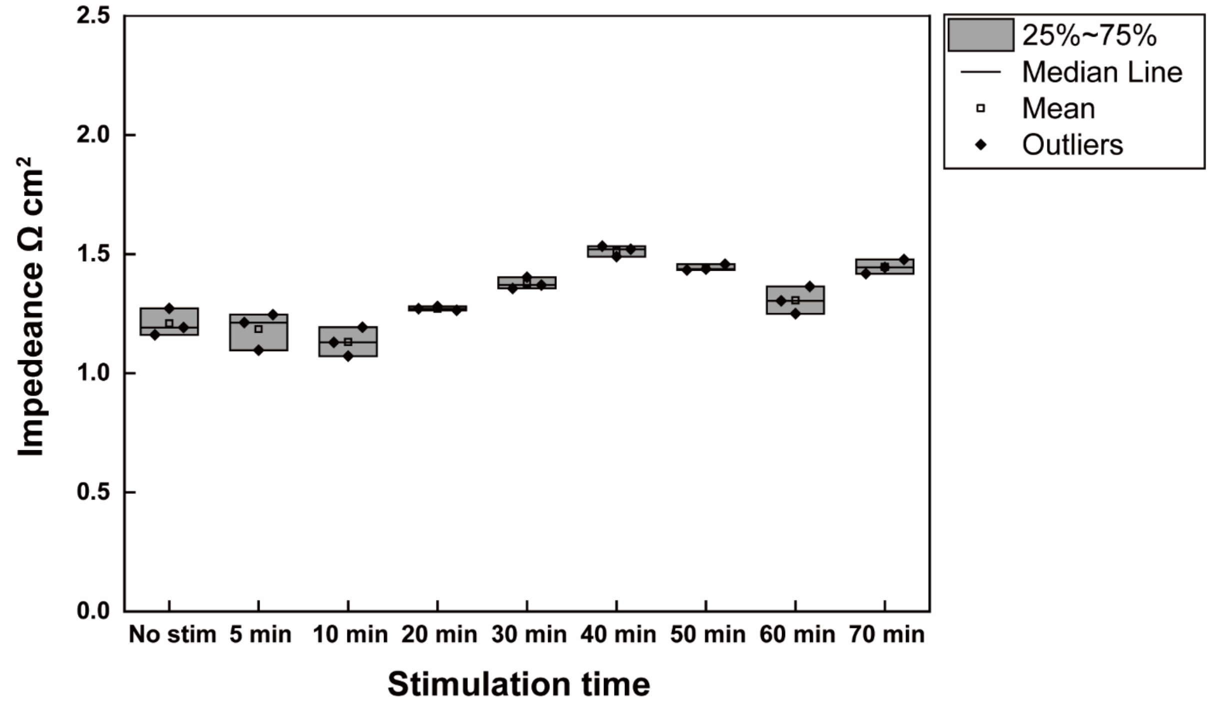

Supplementary Fig. 6. Change in impedance of the electrode as a function of stimulation time.

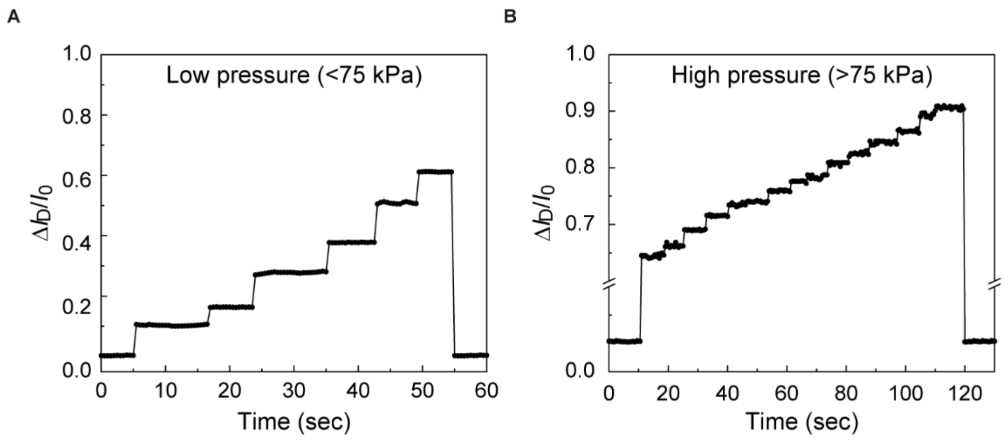

Supplementary Fig. 7. Real-time detection of relative changes in drain current at **(A)** low-pressure ranges (<75 kPa), **(B)** and high-pressure ranges (>75 kPa, <300 kPa). Each step corresponds to 5, 15, 30, 45, 60, 75, 90, 105, 120, 135, 150, 165, 180, 195, 210, 225, 240, 255, 270, 285, and 300 kPa.

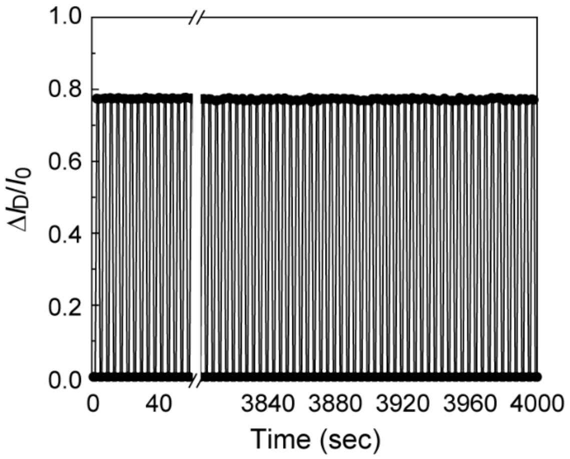

Supplementary Fig. 8. Reliability test during repetitive loading-unloading of pressure at 200 kPa with 1000 cycles.

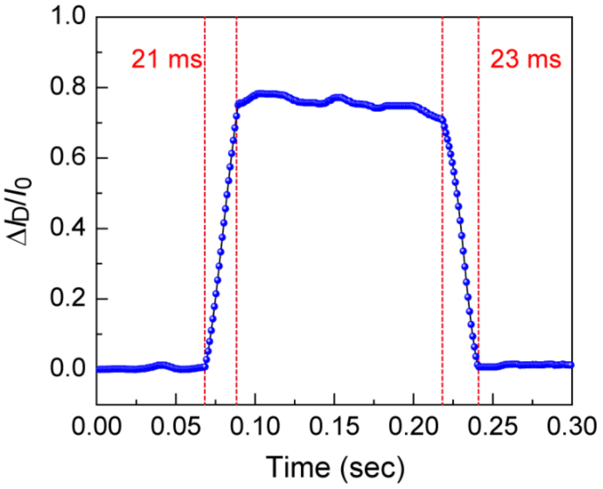

Supplementary Fig. 9. Response time and recovery time during pressure loading at 200 kPa.

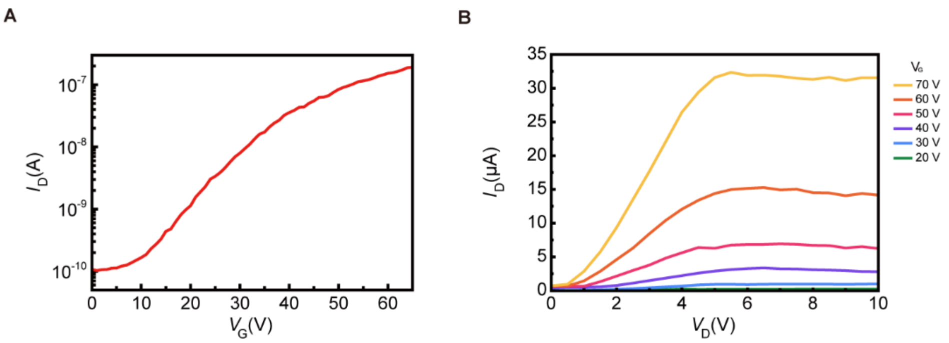

Supplementary Fig. 10. Characteristics of pressure-sensitive FET of TPIEA.

(A) Representative transfer (V_D = 1 V) and **(B)** output (V_G = 20 to 70 V) characteristics.

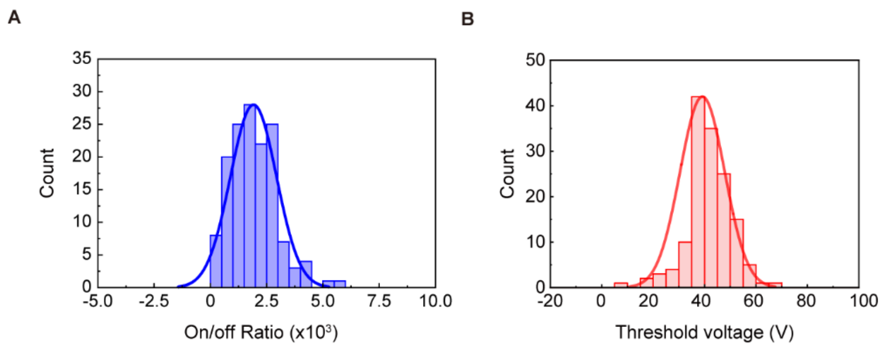

Supplementary Fig. 11. Other characteristics of pressure-sensitive FET of TPIEA.

(A) Statistical distributions of the on/off ratio. **(B)** Threshold voltage of 100 FETs.

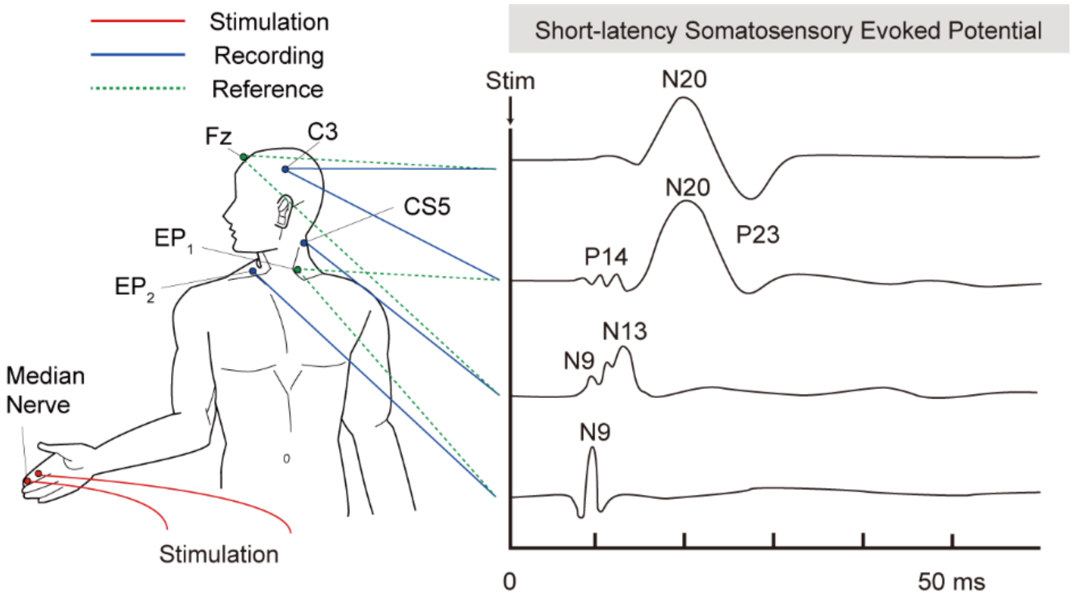

Supplementary Fig. 12. SEP signal peaks according to location of stimulation and recording.

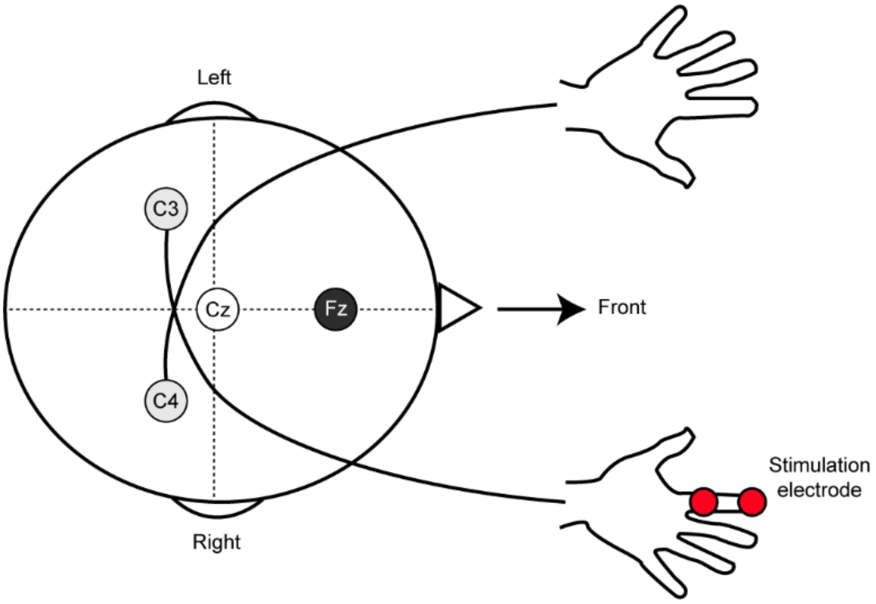

Supplementary Fig. 13. Schematic image showing the positions of the stimulating electrode and recording electrode above.

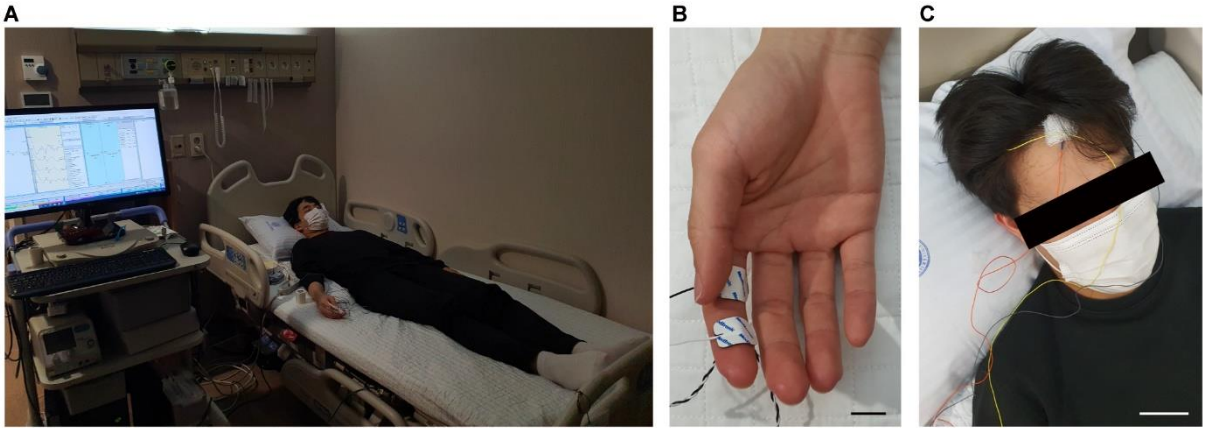

Supplementary Fig. 14. Photographs of SEP experiment with the subject.

(A) Full image. **(B)** Stimulating electrodes on the finger. Scale bars, 1 cm. **(C)** Recording electrodes on C3. Scale bars, 5 cm.

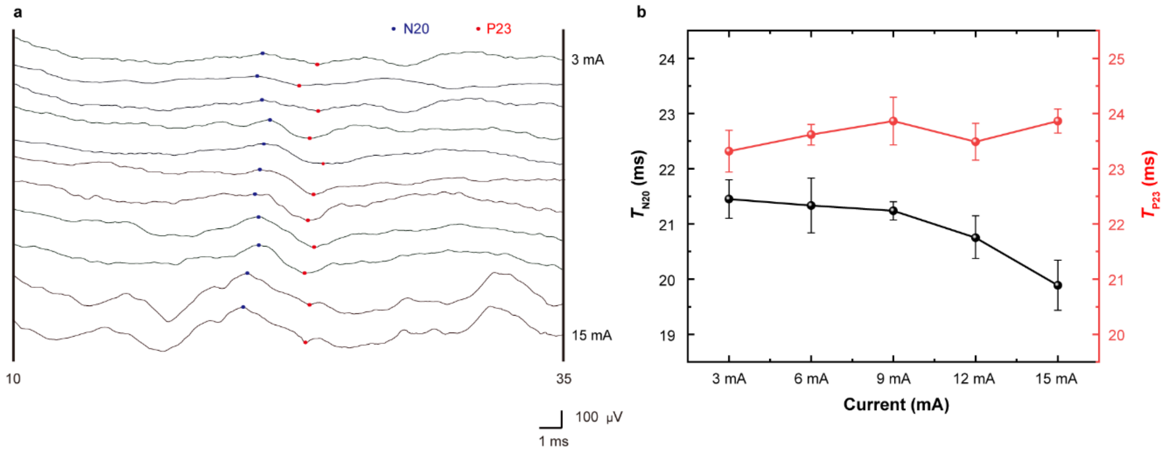

Supplementary Fig. 15. SEP signals according to changes in amplitude.

(A) Raw data of SEP signals measured from 3 mA to 15 mA. **(B)** The post-stimulus peak time of N20 and P23 when current amplitude changed. It showed a low tendency.

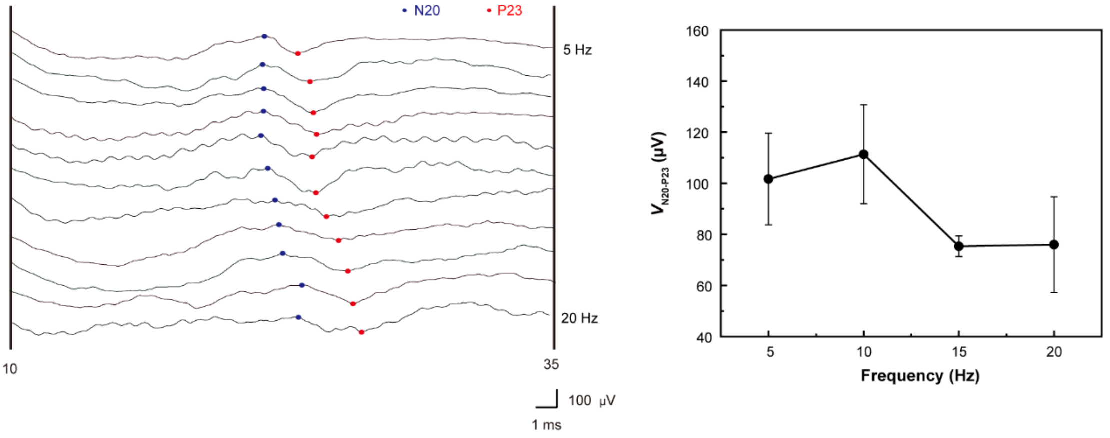

Supplementary Fig. 16. SEP signals according to changes in frequency.

(A) Raw data of SEP signals measured from 5 Hz to 20 Hz **(B)** The peak potential difference of N20 and P23 when current frequency changed. It showed a low tendency.

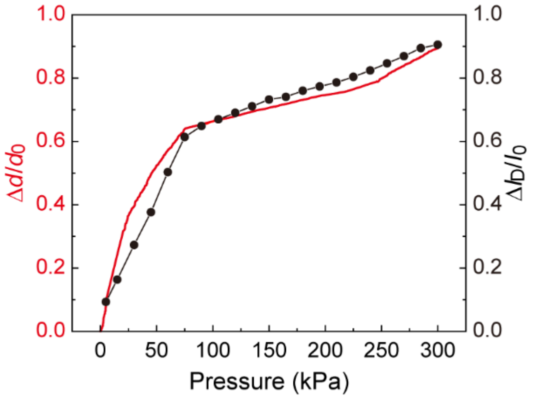

Supplementary Fig. 17. Thickness of an air-dielectric layer (red line) and relative changes in drain current (black) under applied pressure.

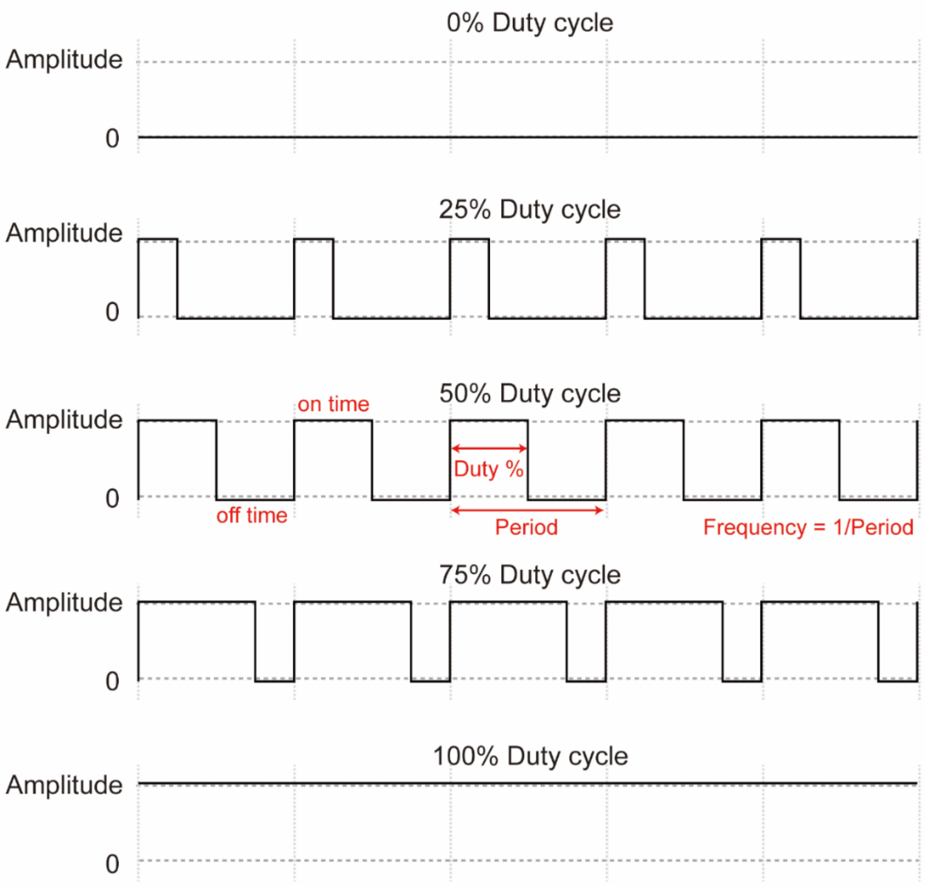

631

632 **Supplementary Fig. 18.** Modulation of pulse width determined by the duty cycle.

633

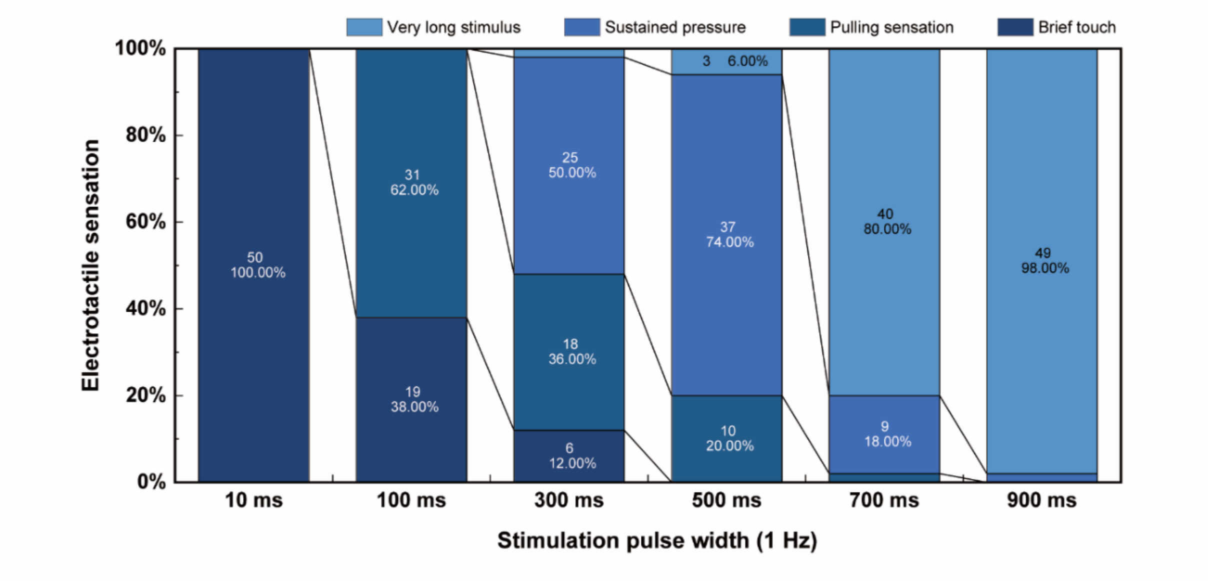

Supplementary Fig. 19. The tendency of the response when the pulse width is 10 ms, 100 ms, 300 ms, 500 ms, 700 ms, and 900 ms. The subjects were encouraged to respond with "Brief touch," "Pulling sensation," "Sustained pressure," or "Very long stimulus." In the response results, it was observed that at a latency of 10 milliseconds, all responses were categorized as "Brief touch." However, there was a shift from "Brief touch" at 100 milliseconds, and with the increment in pulse width, there was an increased tendency to transition from a "Pulling sensation" to "Sustained pressure." Furthermore, starting from 500 milliseconds, there was a increase in the ratio of the "Very long stimulus." Consequently, it was ascertained that a longer pulse width has the capacity to induce a persistent sensation.

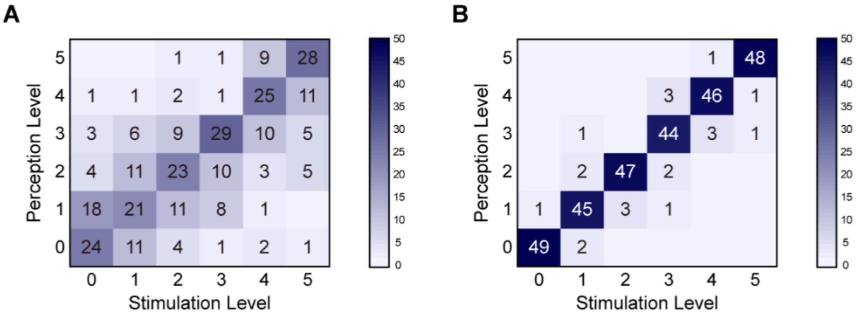

Supplementary Fig. 20. Confusion matrix of perception response with pressure control.

(A) Confusion matrix of perception response to 6 levels of stimulations applied by TPIEA with uncontrolled pressure. **(B)** Confusion matrix of perception response with 100 kPa of controlled pressure.

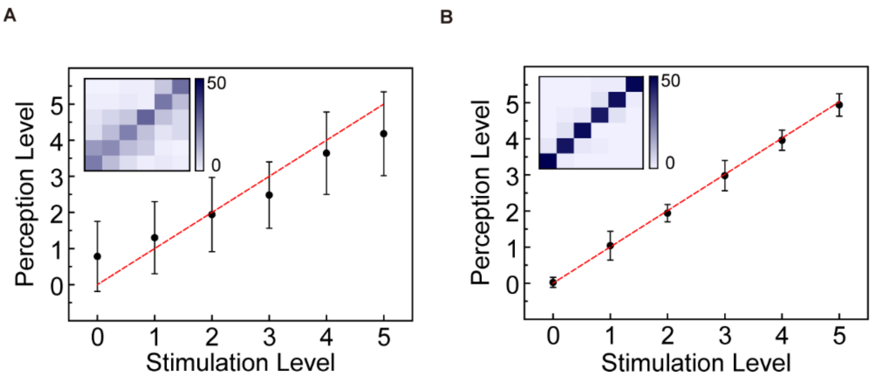

Supplementary Fig. 21. Perception response to the stimulation composed of six levels of current amplitude and confusion matrix (inset) with **(A)** 40 kPa and **(B)** 170 kPa **(B)** of controlled pressure.

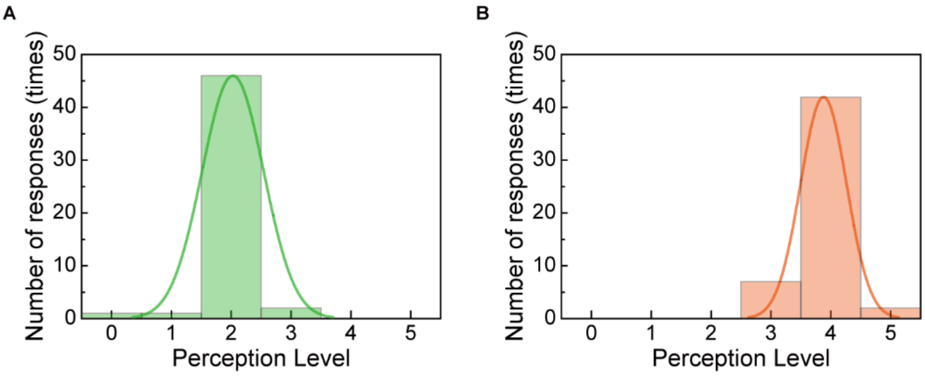

Supplementary Fig. 22. Distribution of responses at a specific stimulation level with a controlled pressure of **(A)** 40 kPa **(B)** and 100 kPa.

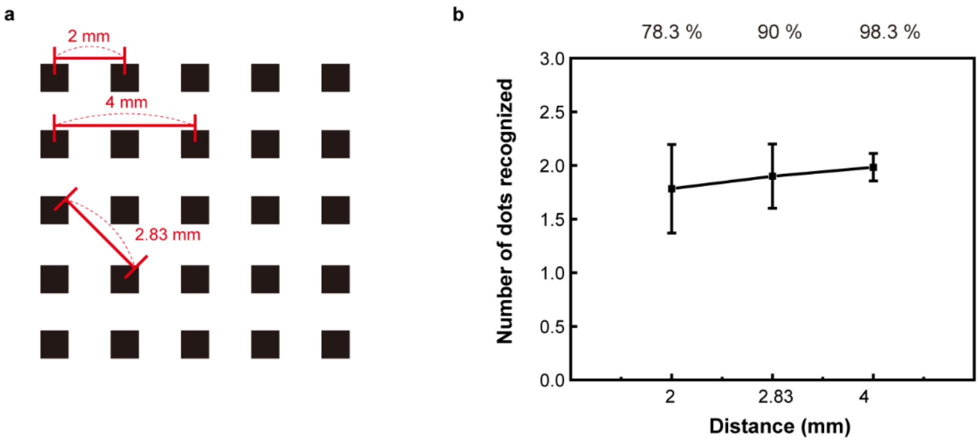

Supplementary Fig. 23. Two-point discrimination experiment.

(A) 2 mm, 2.83 mm, and 4 mm stimulation distances between actuators. **(B)** the result when two-point discrimination was performed 60 times for each distance. It showed 78.3%, 90%, and 98.3% accuracy at 2 mm, 2.83 mm, and 4mm distances respectively.

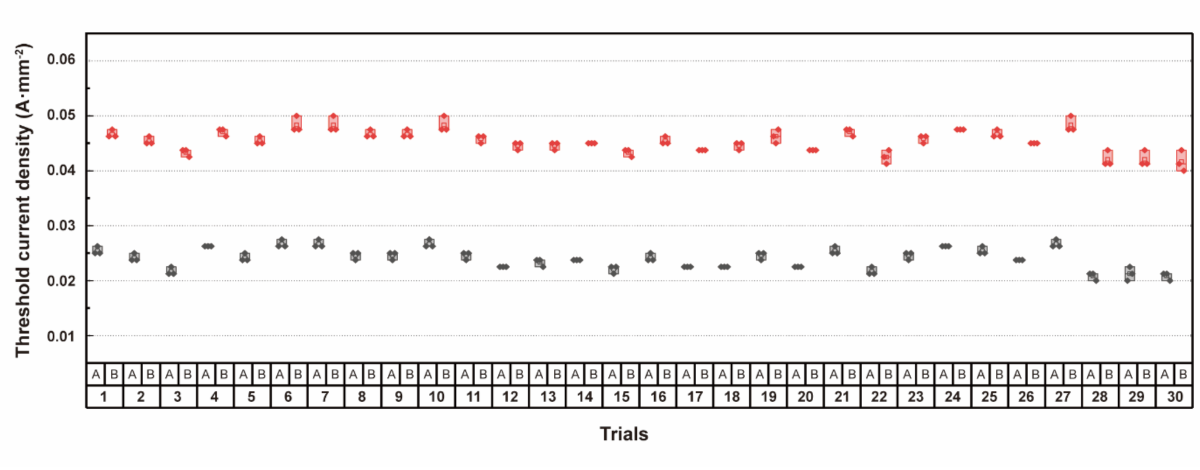

Supplementary Fig. 24. The threshold current density from “Perceptible” level to “Well perceptible” level **(A)** and the threshold current density from “Well perceptible” level to “Very well perceptible” level **(B)** as a result of 30 repeated collections with three subjects.

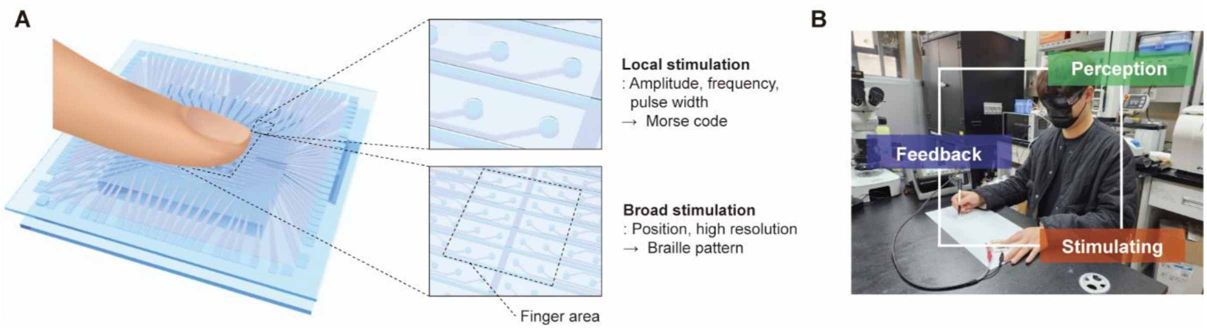

Supplementary Fig. 25. Transferring local and broad tactile information with TPIEA.

(A) Schematic illustration of two types of electrotactile information produced by the TPEA. **(B)** Image of the perception test process. Temporal tactile sensation information can be conveyed by modulating the electrical stimulation in a single electrotactile actuator, whereas spatial tactile sensation information can be conveyed by adjusting the stimulation position of multiple electrotactile actuators. The electrotactile information transfer process through the subject is depicted. TPEA applied to the fingertip of an individual elicits electrotactile stimulation that corresponds to the Morse code or Braille. This process comprises three sequential stages: a stimulation phase, during which electrotactile signals are administered; a perception phase, during which the subject perceives the signals; and a feedback phase.

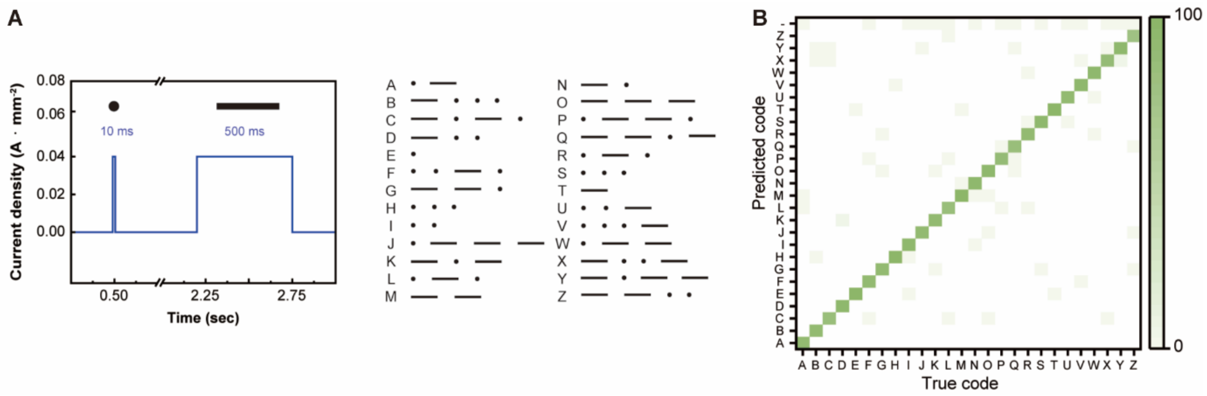

Supplementary Fig. 26. Morse code information via TPIEA.

(A) Stimulation waveform for the Morse code. The dots and dashes in the electrical simulation are distinguished by the pulse width (dot = 10 ms, dash = 500 ms) (left), and letters implemented with a combination of dots and dashes (right). **(B)** Confusion matrix results of 100 trials for Morse code recognition upon finger stimulation using an electrotactile code.

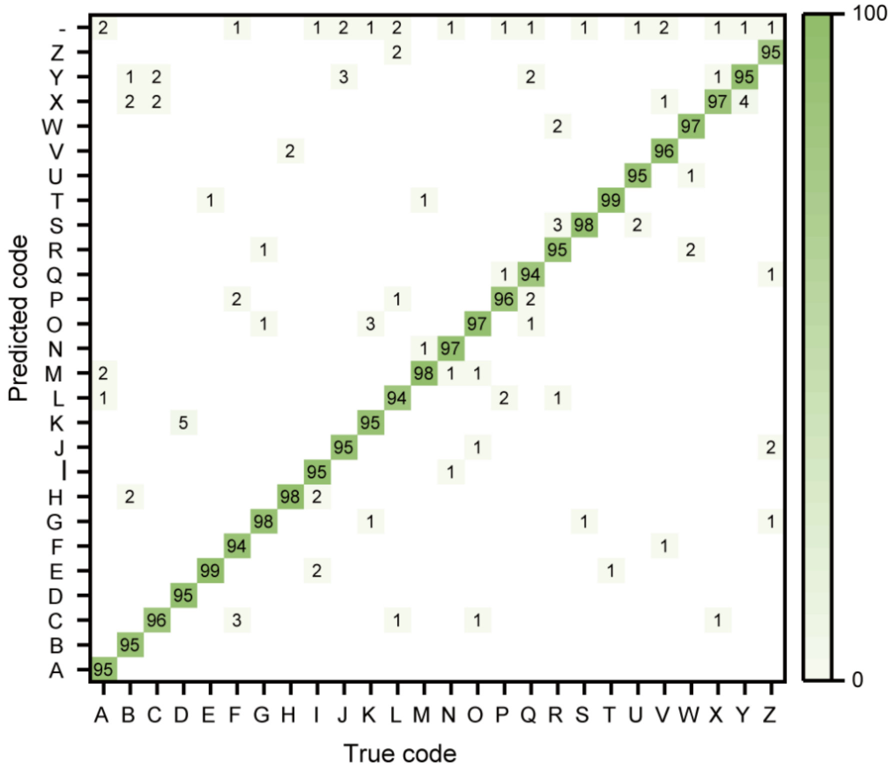

Supplementary Fig. 27. Confusion matrix of perception response with Morse code.

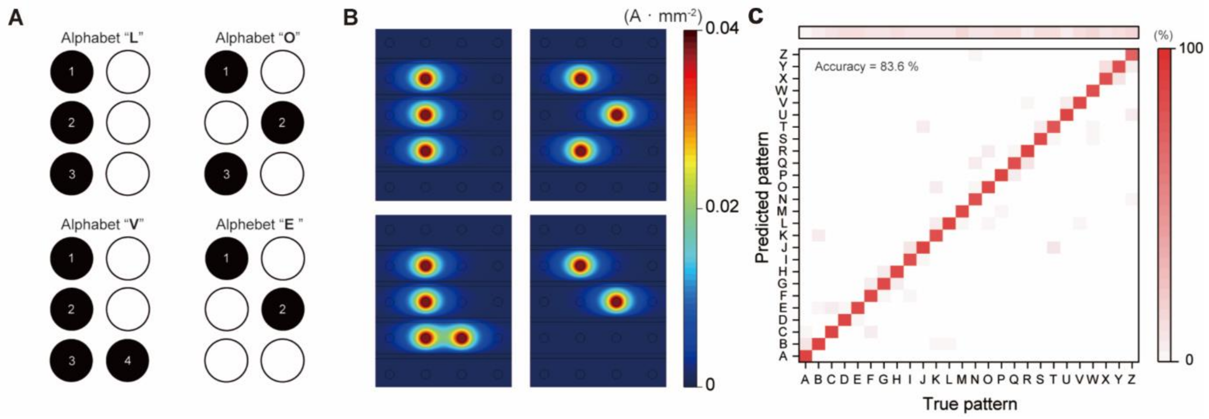

Supplementary Fig. 28. Braille information via TPIEA.

(A) Schematic image of the sequence and number of stimulations for Braille patterns "L," "O," "V," and "E." As the electrical stimulation conditions, the frequency, pulse width, and current density were set to 10 Hz, 10 ms, and 0.04 A mm^{-2}, respectively. (**B**) FEA simulation results of the current density distribution at different Braille patterns "L," "O," "V" and "E." Current-density mapping data when the electrotactile sensation of the Morse code is applied to multiple electrodes. (**C**) Confusion matrix results of 100 trials for Braille recognition upon finger stimulation using electrotactile patterns.

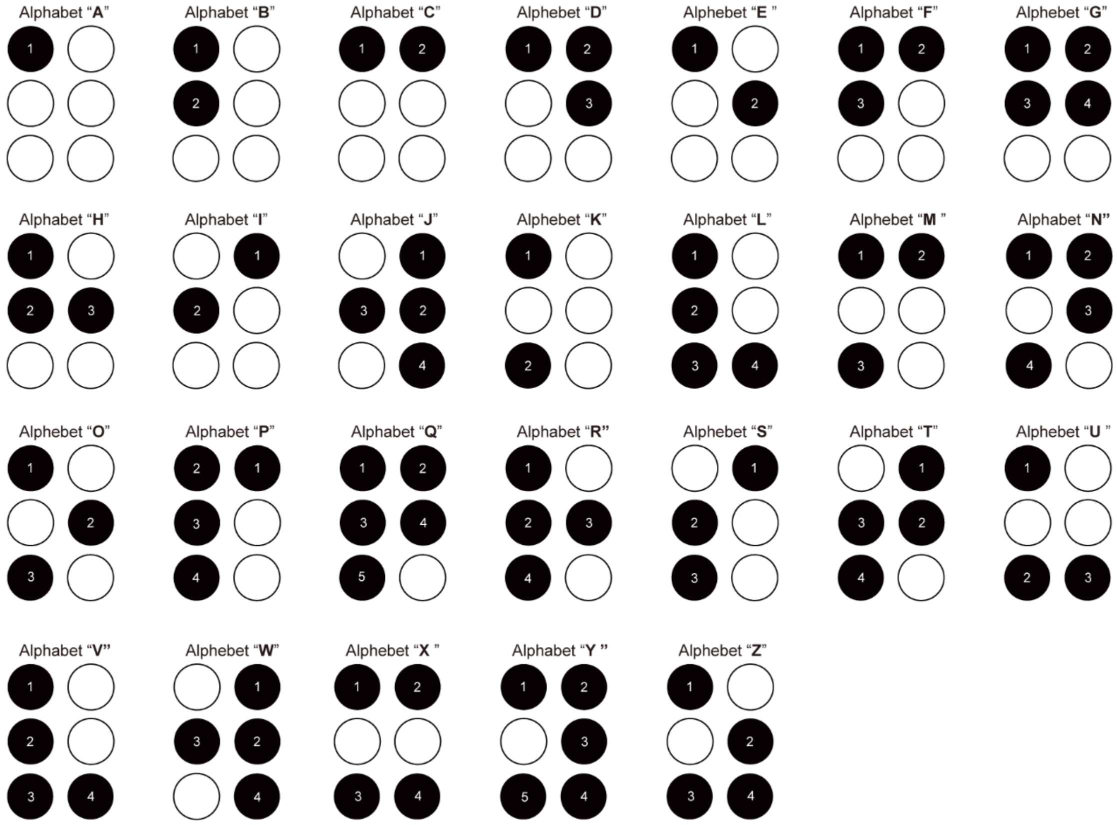

Supplementary Fig. 29. Patterns representing stimulus locations and sequences of braille codes for all alphabets.

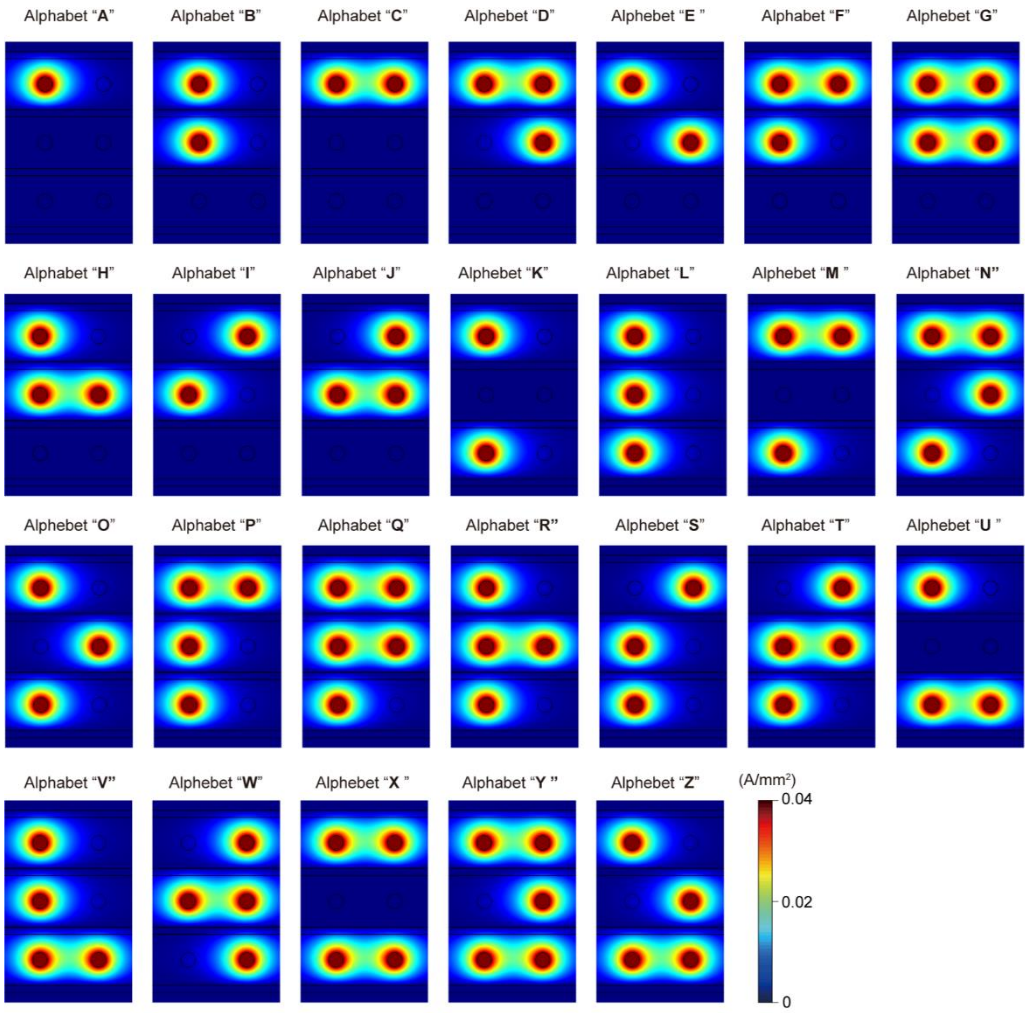

Supplementary Fig. 30. The FEA simulation through the COMSOL Multiphysics program of Fig. S19.

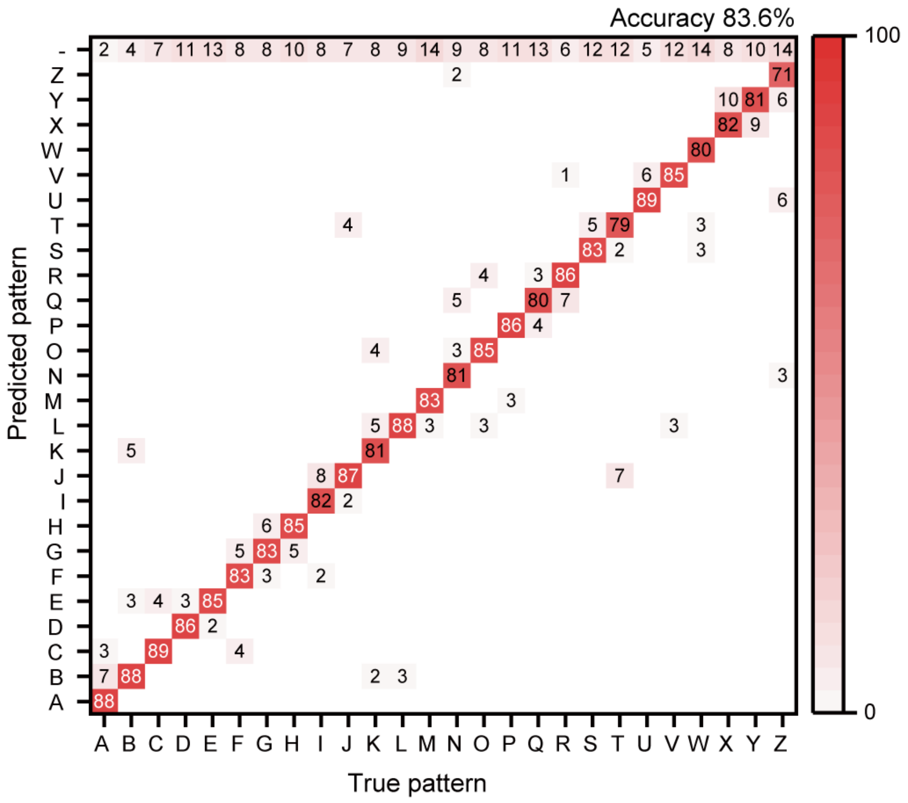

Supplementary Fig. 31. Confusion matrix of perception response with Morse code with 2 mm stimulation distance.

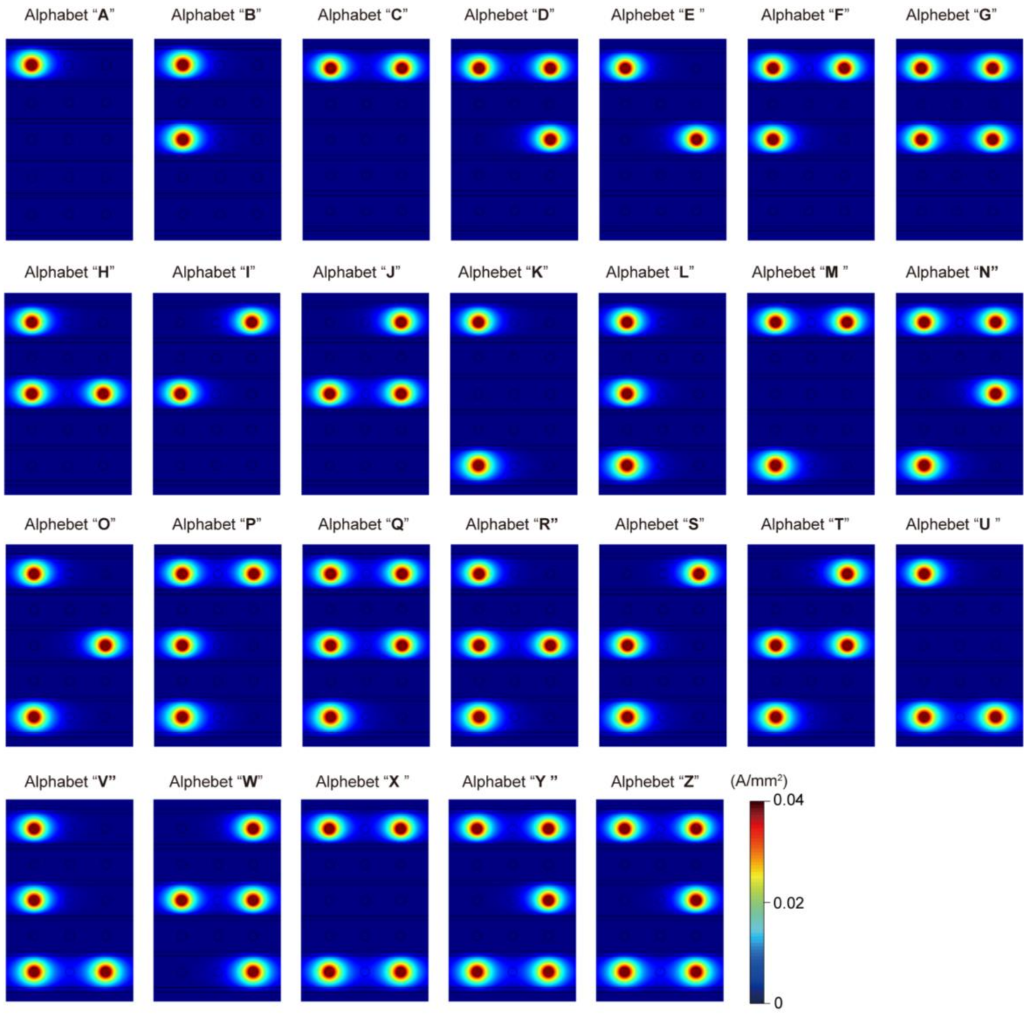

Supplementary Fig. 32. The FEA simulation through the COMSOL Multiphysics program of Fig. S29 when electrode distance is 4 mm.

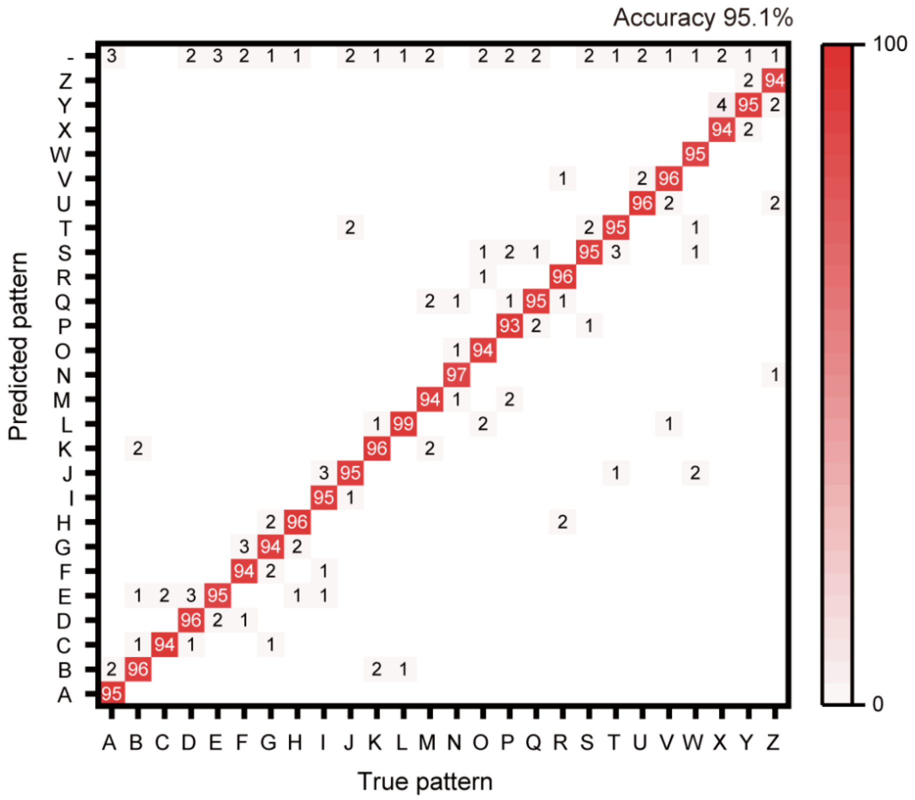

Supplementary Fig. 33. Confusion matrix of perception response with Morse code with 4 mm stimulation distance.

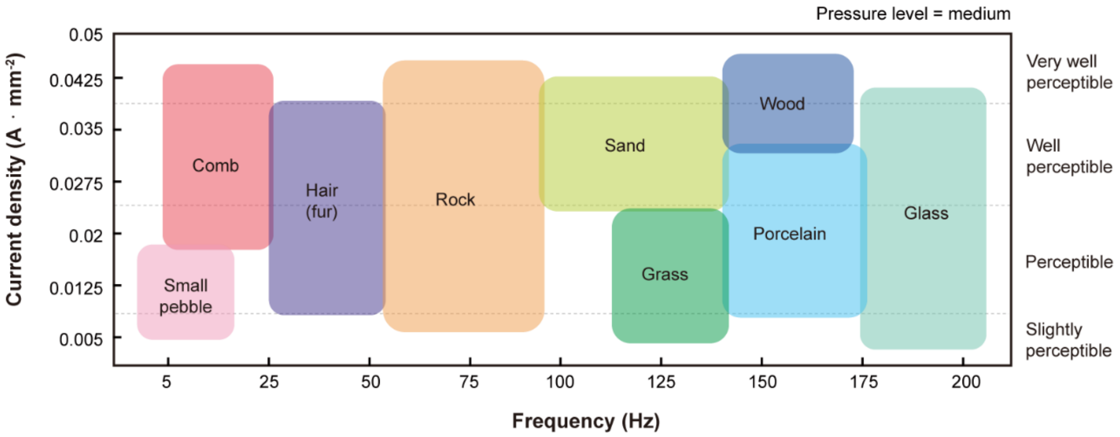

Supplementary Fig. 34. The electro-tactile sensations mimicking map according to frequency and current density.

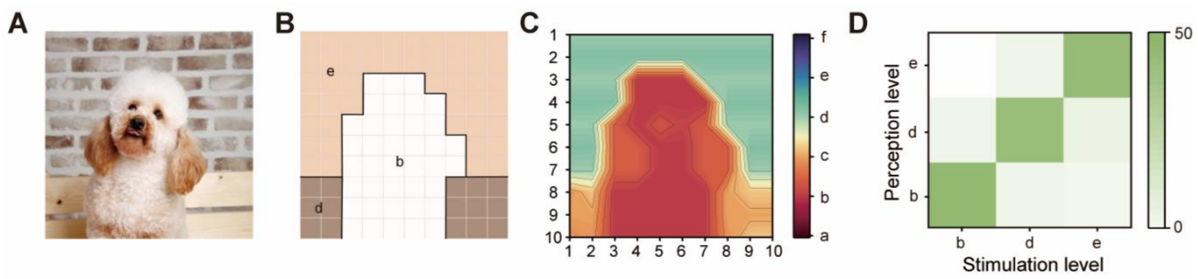

Supplementary Fig. 35. Implementation of virtual tactile sensation for fur, wood, and rock.

(A) Image selected for tactile implementation consisting of fur, wood, and rock (Image source: Gettyimages). **(B)** Schematic image of a 10 × 10 TPIEA array divided for tactile mimicking corresponding to picture. **(C)** Color maps depicting the electrotactile sensation to be implemented according to the divided zones. **(D)** Confusion matrix result of 50 perception test trials corresponding to each electrotactile sensation.

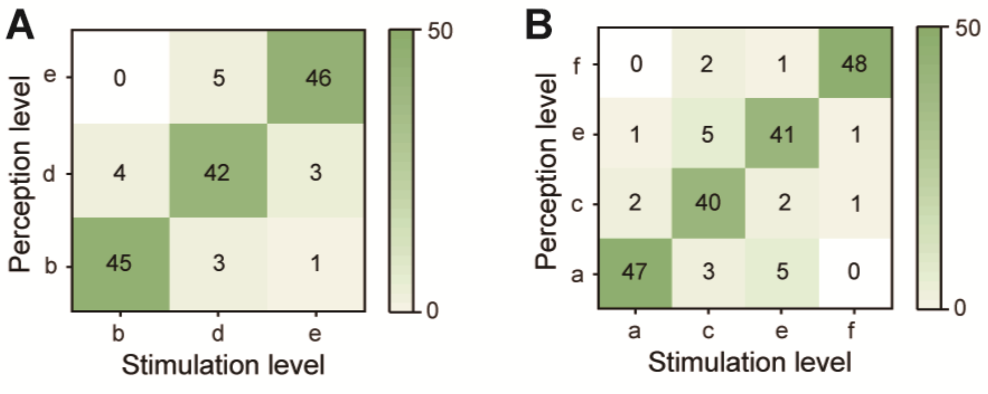

Supplementary Fig. 36. Confusion matrix of perception response with pictures for **(A)** Fig. 4D and **(B)** Fig. S25.

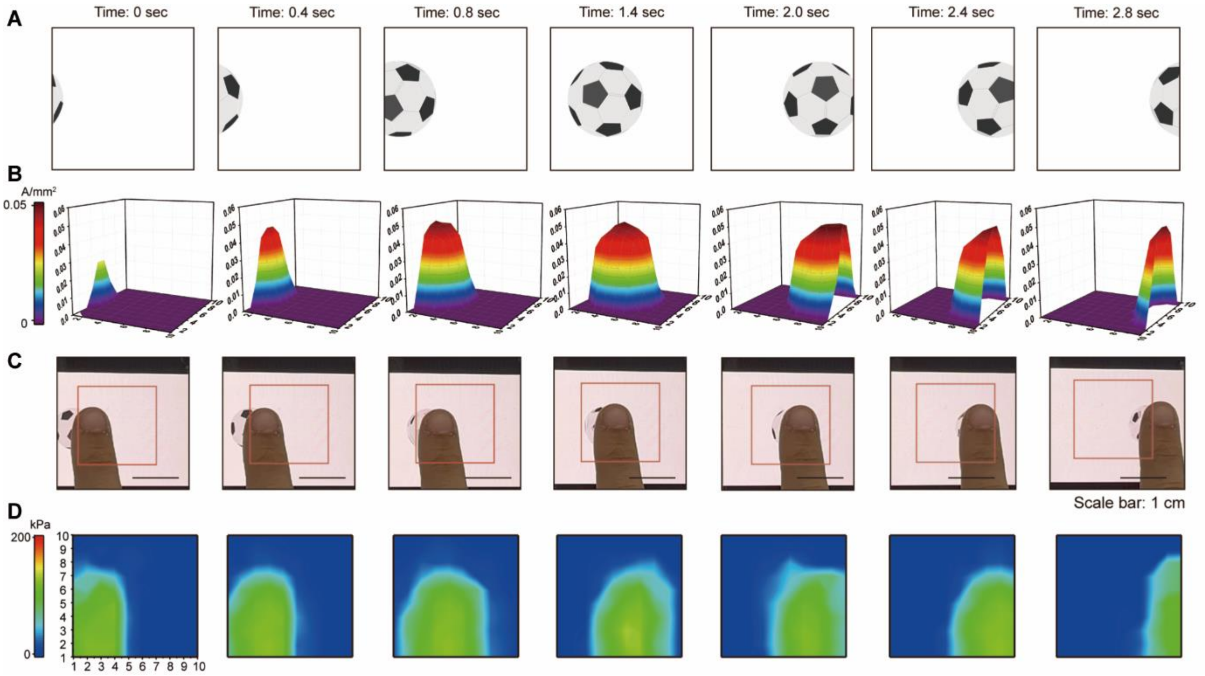

Supplementary Fig. 37. Sequence of TPIEA tactile sensation implementation to follow the movement of a rolling soccer ball.

(A) Images of the rolling ball captured from video. **(B)** Spatiotemporal color maps of the current density of tactile stimulation corresponding to ball movement in 3D. **(C)** Finger movements that follow the direction in which the ball is rolling due to the parallel change in electrotactile sensation implemented. **(D)** Spatiotemporal color maps of finger pressure distribution applied to the display of an electronic device during finger movement from the left to the right side. Video source: Septadi, Adobe Stock.

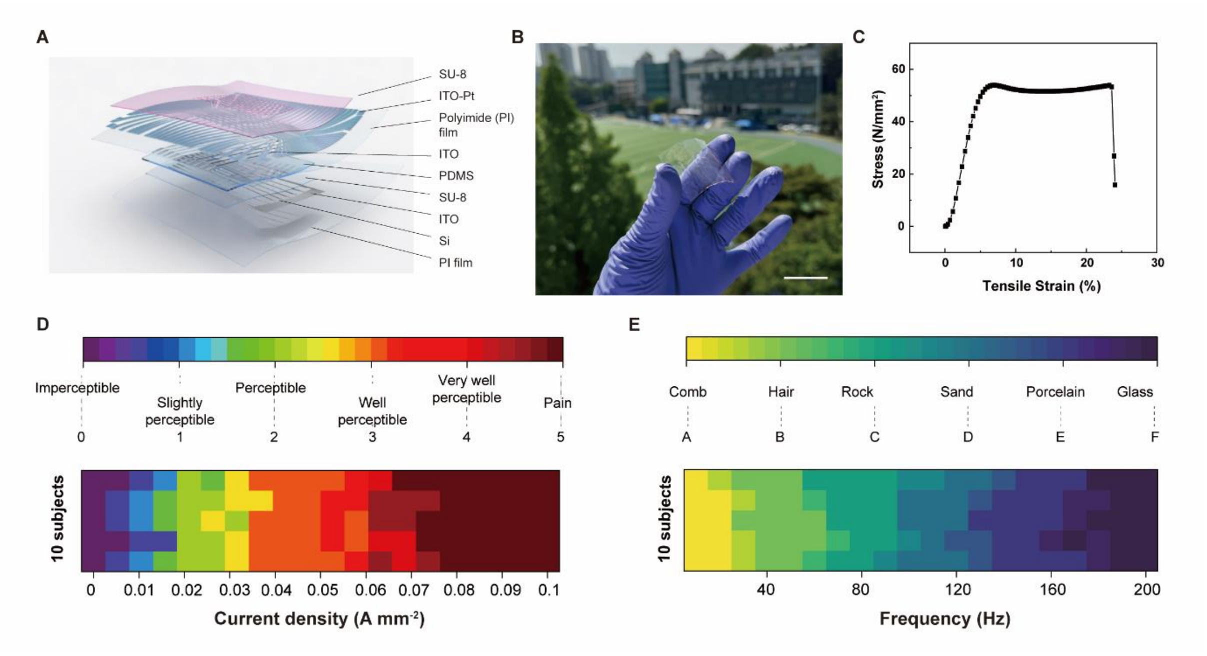

Supplementary Fig. 38. TPIEA device using a flexible polyimide (PI) substrate.

(A) Schematic layouts, and structural designs of the device composed of an electrotactile actuator array and pressure-sensitive transistor array on PI substrate. **(B)** Photograph of the transparent electrotactile actuator with pressure sensor on PI substrate. Scale bar, 2 cm. **(C)** The tensile strength curve of TPIEA with PI substrate, 54.0 MPa. **(D)** Electrotactile intensity for each finger pressure when the current density was changed from 0 to 0.01 A mm^{-2}. Finger pressure was controlled at 100 kPa. **(E)** Electrotactile texture for each finger pressure when the frequency is changed from 0 to 200 Hz.

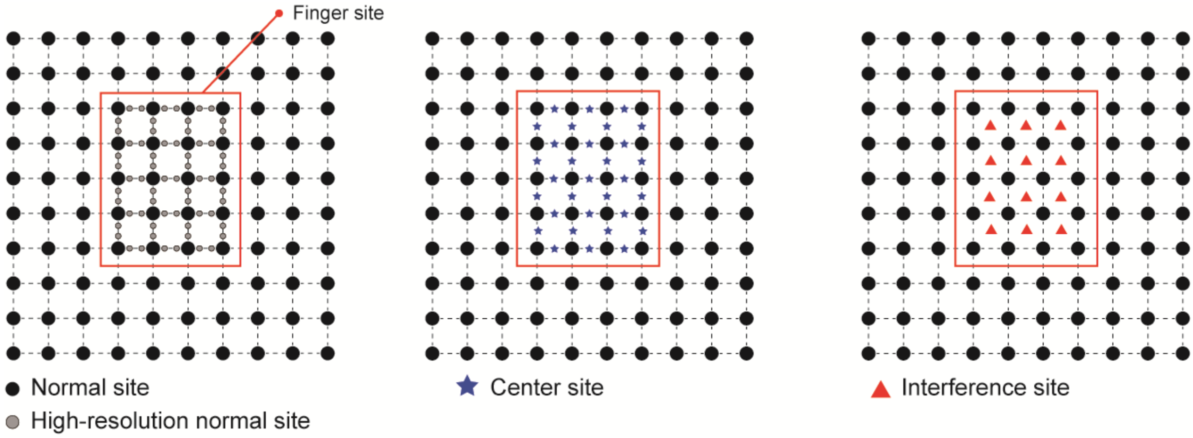

Supplementary Fig. 39. Stimulation sites for various stimulation types. It displays the stimulation sites when a single stimulus is applied using four electrodes (left), when a single stimulus is applied using two electrodes (middle), and when interference stimulus is applied using four electrodes (right).

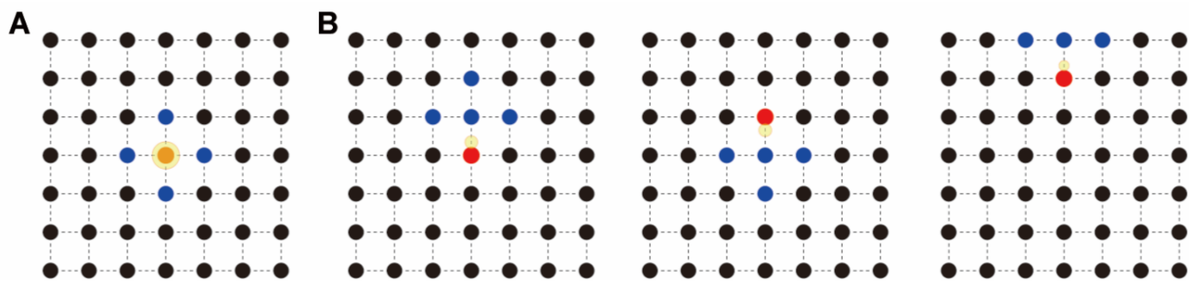

Supplementary Fig. 40. Patterns of a single stimulation site created with "Normal site" and "High-resolution normal site".

The red color represents the anode, the blue color represents the cathode, and the yellow circle represents the stimulation site. **(A)** "Normal site" **(B)** "High-resolution normal site".

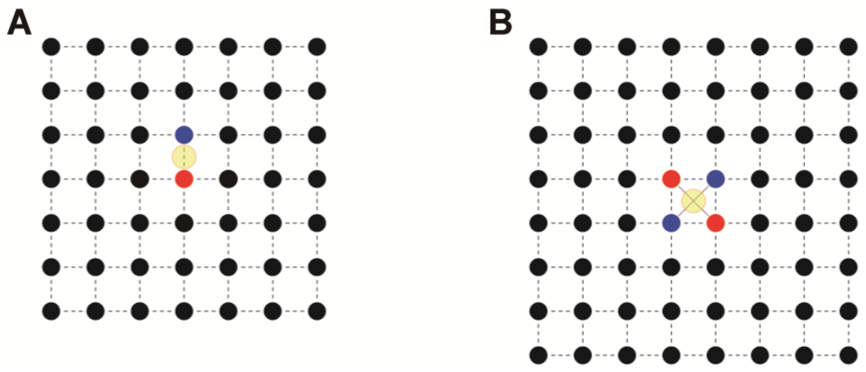

Supplementary Fig. 41. Patterns of "Center site" and "Interference site".

The red color represents the anode, the blue color represents the cathode, and the yellow circle represents the stimulation site. **(A)** "Center site" via single stimulation. **(B)** "Interference site" via interference stimulation.

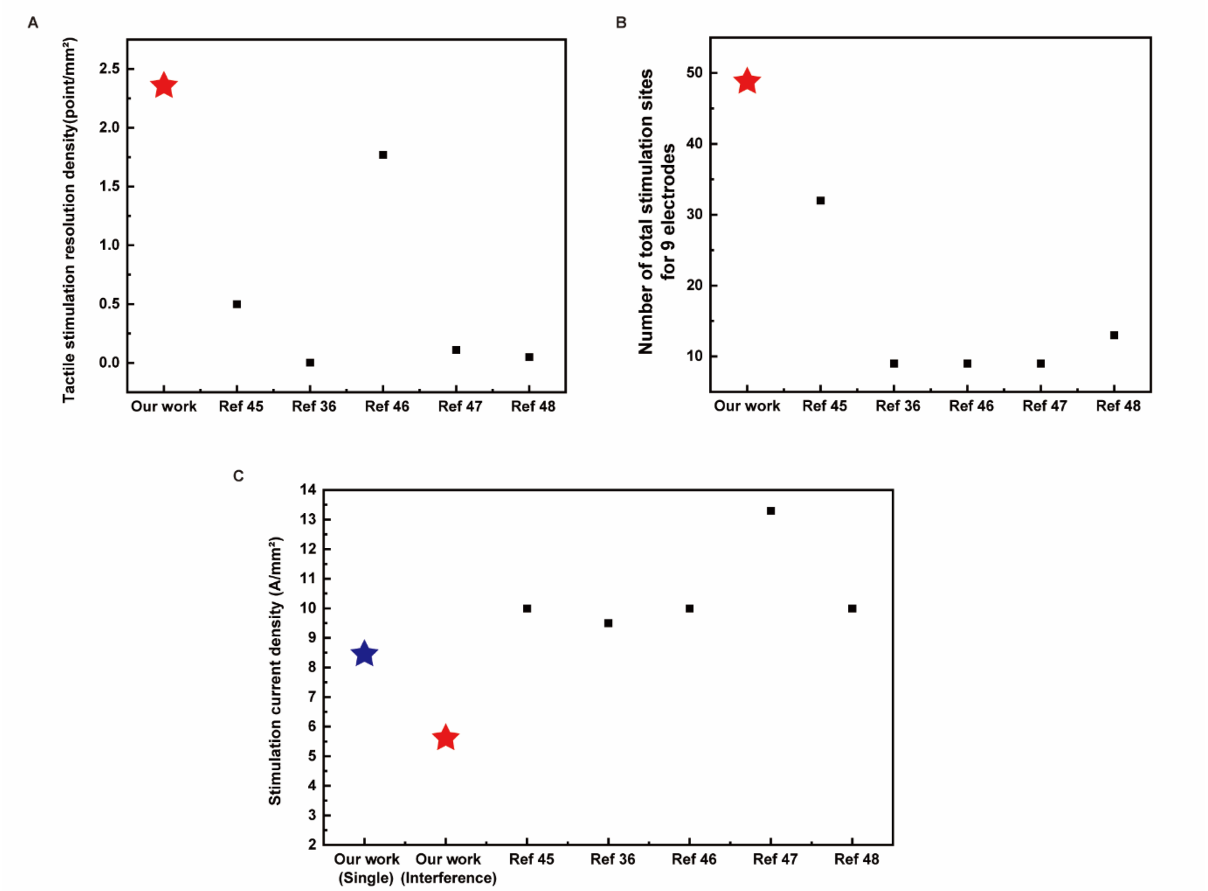

Supplementary Fig. 42. Comparisons of our work with previous studies **(A)** for tactile stimulation density, **(B)** number of total stimulation sites for 9 electrodes, and **(C)** stimulation current density. Blue star is single stimulation and red star is interference stimulation in our work.

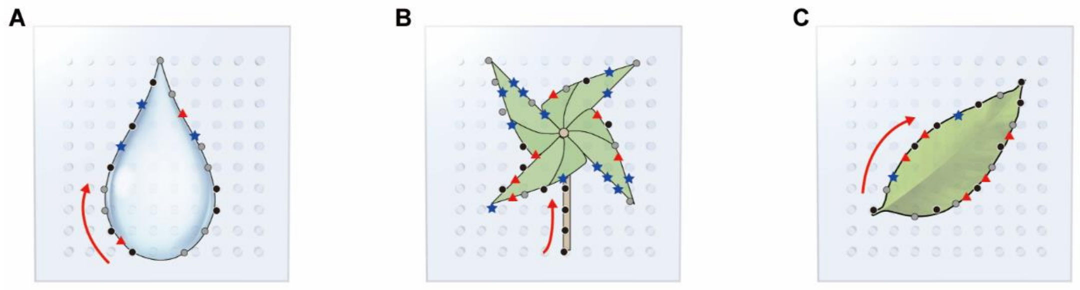

Supplementary Fig. 43. Schematic images of the complex pattern and stimulation sequences created by combining single stimulation sites with interference stimulation sites.

(A) Droplet pattern. **(B)** Pinwheel pattern. **(C)** Leaf pattern.

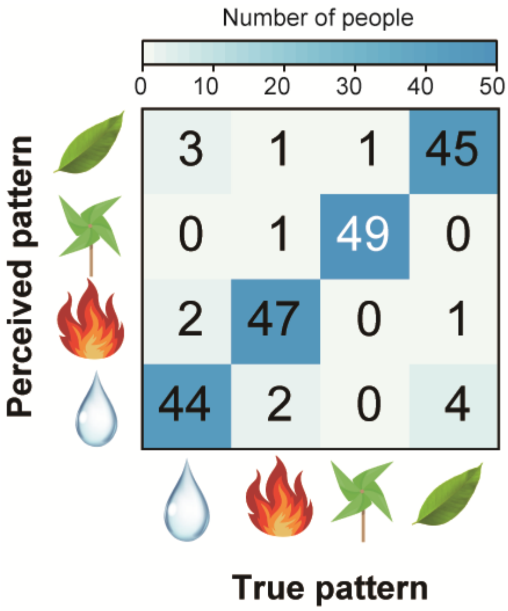

Supplementary Fig. 44. Confusion matrix of perception response with pictures for Fig. 6E.

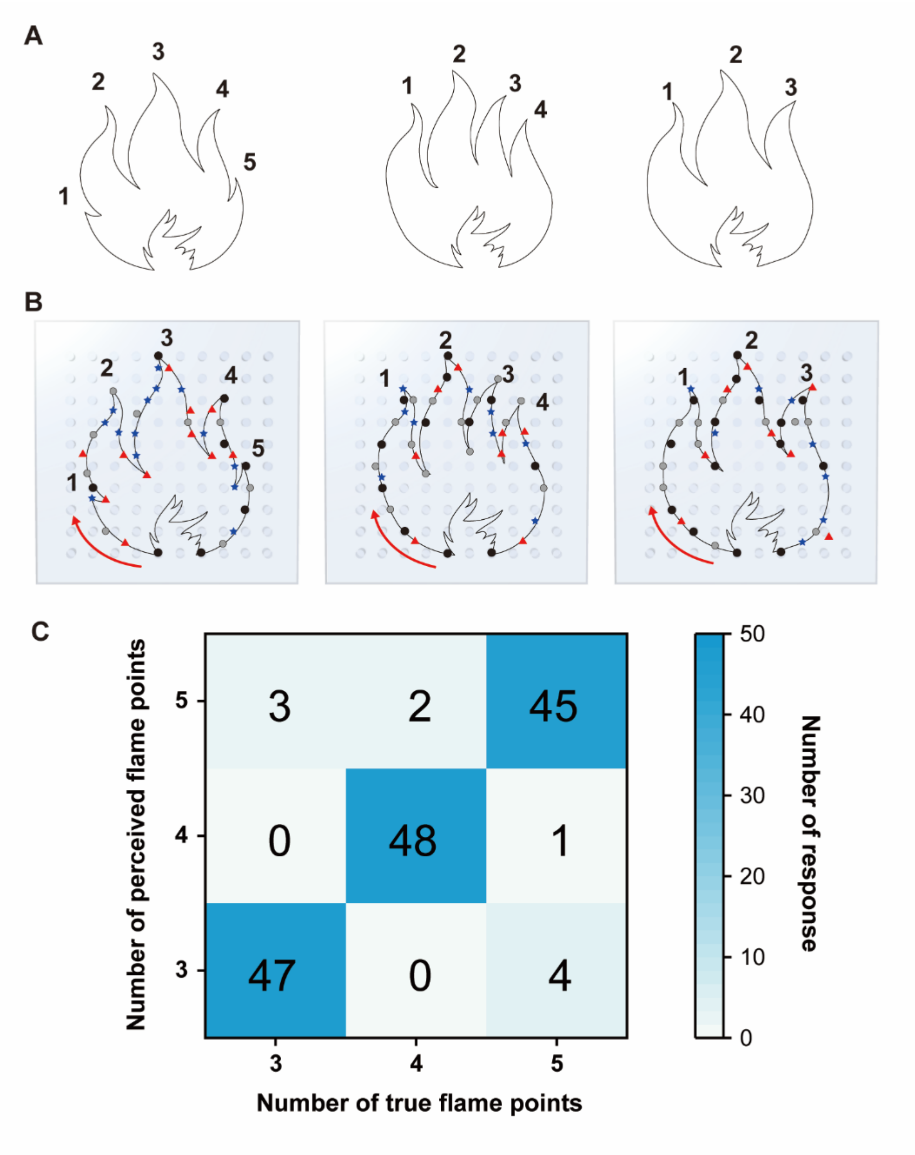

Supplementary Fig. 45. High-resolution interference tactile stimulation that can distinguish between different points in flame patterns.

(A) Three different flame patterns with different numbers of prepared points: 5 points, 4 points, 3 points. **(B)** Schematic images of the complex pattern and stimulation sequence of a flame created by combining single stimulation sites with interference stimulation sites. The number of points in the flame is 5, 4, and 3 from left to right. **(C)** Confusion matrix results of 50 perception trials corresponding to each pattern. The results indicated an accuracy rate of 93.4%.

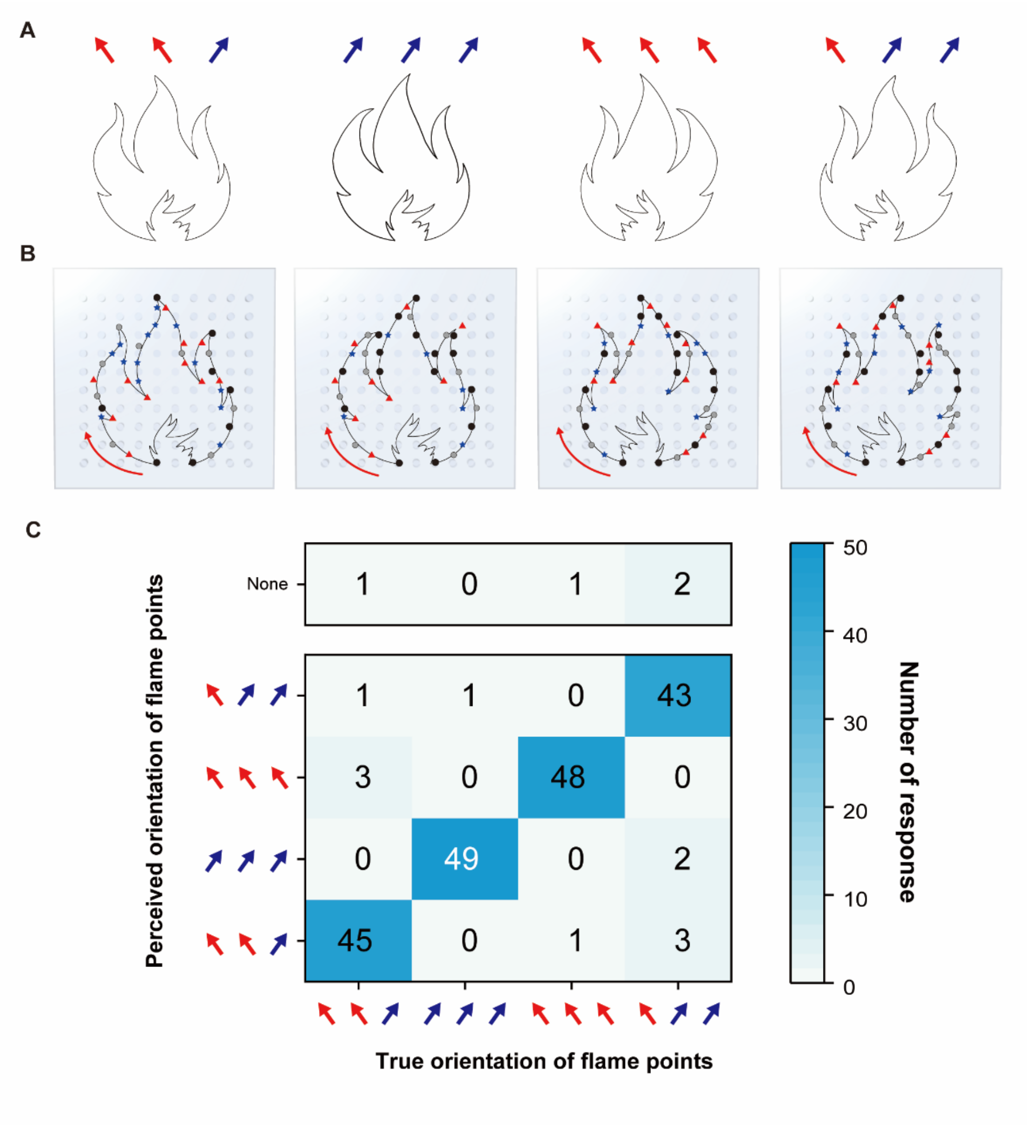

Supplementary Fig. 46. High-resolution interference tactile stimulation that can distinguish between different directions of flames.

(A) Four different patterns of the main fireworks directions: (left, left, right), (right, right, right), (left, left, left), (left, right, right). **(B)** Schematic images of the complex pattern and stimulation sequence of a flame created by combining single stimulation sites with interference stimulation sites which is distinguished in directions of flame. **(C)** Confusion matrix results of 50 perception trials corresponding to each pattern. The findings revealed an accuracy rate of 92.5%.

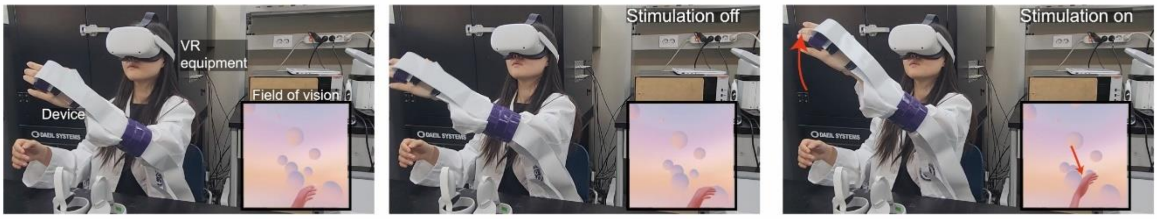

Supplementary Fig. 47. Electrotactile sensation application through TPIEA with VR equipment.

Supplementary Table

Name	Location	Depth	Sensation	Frequency
Slowly adapting type 1(SA1)	Merkel disk end-organ	0.9 mm	Static force, skin indentation, fine discrimination of textures.	0.4 – 10 Hz
Slowly adapting type 2(SA2)	Ruffini corpuscle end-organ	-	Stretching, pressure.	0.4 – 10 Hz
Rapidly Adapting type 1(RA1)	Meissner corpuscle end-organ	0.7 mm	Low frequency vibration, movement of skin texture.	50 Hz – 120 Hz
Rapidly Adapting type 2(RA2)	Pacinian corpuscle end-organ	~2.0 mm	High frequency vibration.	200 – 500 Hz
Free nerve ending	Free nerve ending	-	Touch, pressure, stretching, temperature, itch.	-

Supplementary Table 1. Classification of locations, responsible senses, and stimulation frequency according to each mechanoreceptor.

Sensor's Type	Detection target	Detection Range	Response time	Array size	Ref.
Capacitive	Finger pressure	~ 100 kPa	190 ms	1	37
	Various human motions	0 ~ 600 kPa	280 ms	1	38
	Eye movement	5 ~ 40°	-	4	39
Piezoelectric	Wrist pulse	0.125 ~ 22.5 kPa	18.6 ms	3	40
	Arterial pulse	1 ~ 60 kPa	60 ms	1	41
	Finger pressure	0 ~ 25 kPa	300 ms	9 (3 × 3)	42
Pressure-sensitive transistor	Wrist pulse	8 ~ 1500 Pa	< 10 ms	25 (5 × 5)	43
	Cellular motion	200 Pa ~ 5 MPa	25 ms	400 (20 × 20)	44
	Finger pressure (this work)	5 ~ 300 kPa	21 ms	100 (10 × 10)	-

Supplementary Table 2. Comparison in performances of three types of pressure sensors utilized for motion detection.

<table>
<tr><th></th><th>Tactile stimulation resolution density (points mm^{-2})</th><th>Number of total simulation sites for 9 electrodes</th><th colspan="2">Stimulation current density (A mm^{-2})</th><th>Ref</th></tr>
<tr><td>This work</td><td>2.37</td><td>49</td><td>8.5</td><td>5.6</td><td>-</td></tr>
<tr><td>Science Advances 8 (36), eabp8738 (2022).</td><td>0.5</td><td>32</td><td colspan="2">10</td><td>[45]</td></tr>
<tr><td>Science Advances, 7(6), eabe2943 (2021).</td><td>0.0025</td><td>9</td><td colspan="2">9.5</td><td>[36]</td></tr>
<tr><td>IEEE Transactions on Haptics, 5(2), 184-188 (2012).</td><td>1.77</td><td>9</td><td colspan="2">10</td><td>[46]</td></tr>
<tr><td>IEEE Transactions on Biomedical Engineering, 46(8), 929-936 (1999).</td><td>0.11</td><td>9</td><td colspan="2">13.3</td><td>[47]</td></tr>
<tr><td>ACS omega, 3(1), 662-666 (2018).</td><td>0.05</td><td>13</td><td colspan="2">10</td><td>[48]</td></tr>
</table>

Supplementary Table 3. Comparison of the tactile resolution and stimulation current density of our work with other previous studies.

Material	Chemical formula	Characteristics		Fabrication method	Supplier
Indium Tin Oxide	In_2O_3-SnO_2 (90:10 wt%)	Transmission range	400-1,000 nm	Sputtering	DasomRMS
		Evaporation temperature	1450 ℃		
		Purity	99.9%		
Polydimethylsiloxane	$CH_3[Si(CH_3)_2O]_n Si(CH_3)_3$	Dielectric constant	~2.7	Curing	Dow
		Viscosity	3000-10,000 $N \cdot s/m^2$		
Lead acetate trihydrate	$Pb(CH_3CO_2)_2 \cdot 3H_2O$	Molecular weight	379.33 g/mol	Electroplating	Sigma-Aldrich
		Purity	99.99%		
Platinum tetrachloride	$PtCl_4$	Molecular weight	336.89 g/mol	Electroplating	Sigma-Aldrich
		Purity	96%		

Material	Usage	Thickness	Fabrication method	Supplier
SU-8 2002	Passivation layer	2-100 μm	Spin coating	MicroChem
LOR 3A	Sacrificial layer	250-600 nm	Spin coating	KAYAKU
S1818	Photoresist for patterning	1.5-2.7 μm	Spin coating	Dow Chemical

Supplementary Table 4. Table organizing the materials used in the experiment.

Supplementary Video

Supplementary Video 1. Tactile information transmission through TPIEA.

Supplementary Video 2. Display-integrated electrotactile sensation through TPIEA.

Supplementary Video 3. Interference stimulation sensation perception test.

Supplementary Reference

1. Kajimoto, H. Electro-tactile Display: Principle and Hardware. *Pervasive Haptics: Science, Design, and Application* (Springer Japan, Tokyo, 2016)
2. Pamungkas, D. S. & Caesarendra, W. Overview Electrotactile Feedback for Enhancing Human Computer Interface. *J. Phys. Conf. Ser.* **1007**, 012001 (2018).
3. Luo. L. *Principles of Neurobiology*. (Garland Science, New York, 2020)
4. Hill, R. W., Wyse, G. A. & Anderson, M. *Animal Physiology* (MA: Sinauer Associates, Sunderland, 2012)
5. Kono, M., Takahashi, T., Nakamura, H., Miyaki, T. & Rekimoto, J. Design Guideline for Developing Safe Systems that Apply Electricity to the Human Body. *ACM Trans. Comput.-Hum. Interact.* **25**, 1-36(2018).
6. IEC 2016. IEC 60479-1: Effects of Current on Human Beings and Livestock. IEC.
7. IEC 2012. IEC 60601-2-10: The requirements for the safety and essential performance of nerve and muscle stimulators, for use in the practice of physical medicine. IEC.
8. JIS 2003. Electric Therapy Apparatus for Home Use. JIS T 2003:2011.
9. JIS 2009. Safety of Household and Similar Electrical Appliances – Part 2-209: Particular Requirements for Electric Therapy Apparatus for Home Use. JIS C 9335-2-209.
10. Cabanes, J. *Electrical Shock Safety Criteria Ch.1* (Pergamon, Oxford, 1985).
11. Rollman, G. B. & Harris, G. The detectability, discriminability, and perceived magnitude of painful electrical shock. *Percept. Psychophys.* **42**, 257–268 (1987).
12. Kritchevsky, M. & Wiederholt, W. C. Short-latency somatosensory evoked potentials. *Arch. Neurol.* **35**, 706–711 (1978).
13. Macerollo, A., Brown, M. J. N., Kilner, J. M. & Chen, R. Neurophysiological Changes Measured Using Somatosensory Evoked Potentials. *Trends Neurosci.* **41**, 294–310 (2018).

14. Kalogianni, K., Daffertshofer, A., van der Helm, F. C. T., Schouten, A. C. & de Munck, J. C. Disentangling Somatosensory Evoked Potentials of the Fingers: Limitations and Clinical Potential. *Brain Topogr.* **31**, 498–512 (2018).
15. Tjepkema-Cloostermans, M. C., van Putten, M. J. A. M. & Horn, J. Prognostic Use of Somatosensory Evoked Potentials in Acute Consciousness Impairment. *Clinical Neurophysiology in Disorders of Consciousness: Brain Function Monitoring in the ICU and Beyond* (Springer, Vienna, 2015).
16. Maudrich, T., Hähner, S., Kenville, R. & Ragert, P. Somatosensory-Evoked Potentials as a Marker of Functional Neuroplasticity in Athletes: A Systematic Review. *Front. Physiol.* **12**, (2022).
17. Tominaga, M. & Julius, D. Capsaicin Receptor in the Pain Pathway. *Jpn. J. Pharmacol.* **83**, 20–24 (2000).
18. DL, W. Pain in children: Comparison of assessment scales. *Pediatr Nurs* **14**, 9–17 (1988).
19. Özgül, Ö. S. *et al.* High test-retest-reliability of pain-related evoked potentials (PREP) in healthy subjects. *Neurosci. Lett.* **647**, 110–116 (2017).
20. Bruyns-Haylett, M. *et al.* The neurogenesis of P1 and N1: A concurrent EEG/LFP study. *NeuroImage* **146**, 575–588 (2017).
21. Cauller, L. J. & Kulics, A. T. The neural basis of the behaviorally relevant N1 component of the somatosensory-evoked potential in SI cortex of awake monkeys: evidence that backward cortical projections signal conscious touch sensation. *Exp. Brain Res.* **84**, (1991).
22. Lu, F. *et al.* Review of Stratum Corneum Impedance Measurement in Non-Invasive Penetration Application. *Biosensors* **8**, 31 (2018).
23. Yokus, M. A. & Jur, J. S. Fabric-Based Wearable Dry Electrodes for Body Surface Biopotential Recording. *IEEE Trans. Biomed. Eng.* **63**, 423–430 (2016).

24. Jayaraman, A., Kaczmarek, K. A., Tyler, M. E. & Okpara, U. O. Effect of localized ambient humidity on electrotactile skin resistance. *2007 IEEE 33rd Annual Northeast Bioengineering Conference* 110–111 (2007).
25. Li, D. *et al.* Non-invasive measurement of normal skin impedance for determining the volume of the transdermally extracted interstitial fluid. *Measurement* **62**, 215–221 (2015).
26. Engebretsen, K. a., Johansen, J. d., Kezic, S., Linneberg, A. & Thyssen, J. p. The effect of environmental humidity and temperature on skin barrier function and dermatitis. *J. Eur. Acad. Dermatol. Venereol.* **30**, 223–249 (2016).
27. Tong, J., Mao, O. & Goldreich, D. Two-Point Orientation Discrimination Versus the Traditional Two-Point Test for Tactile Spatial Acuity Assessment. *Front. Hum. Neurosci.* **7**, 579 (2013).
28. Gellis, M. & Pool, R. Two-point discrimination distances in the normal hand and forearm: application to various methods of fingertip reconstruction. *Plast. Reconstr. Surg.* **59**, 57–63 (1977).
29. Nolan, M. F. Two-Point Discrimination Assessment in the Upper Limb in Young Adult Men and Women. *Phys. Ther.* **62**, 965–969 (1982).
30. Hutcheon, B. *et al.* Resonance, oscillation and the intrinsic frequency preferences of neurons. *Trends Neurosci.* **23**, 216–222 (2000).
31. Mirzakhalili, E., Barra, B., Capogrosso, M. & Lempka, S. F. Biophysics of Temporal Interference Stimulation. *Cell Syst.* **11**, 557-572 (2020).
32. Grossman, N. *et al.* Noninvasive Deep Brain Stimulation via Temporally Interfering Electric Fields. *Cell* **169**, 1029-1041 (2017).
33. Hayward, V., Astley, O. R., Cruz-Hernandez, M., Grant, D. & Robles-De-La-Torre, G. Haptic interfaces and devices. *Sens. Rev.* **24**, 16–29 (2004).

34. Strong, R. M. & Troxel, D. E. An Electrotactile Display. *IEEE Trans. Man-Mach. Syst.* **11**, 72–79 (1970).
35. Kourtesis, P., Argelaguet, F., Vizcay, S., Marchal, M. & Pacchierotti, C. Electrotactile Feedback Applications for Hand and Arm Interactions: A Systematic Review, Meta-Analysis, and Future Directions. *IEEE Trans. Haptics* **15**, 479–496 (2022).
36. Shi, Y. *et al.* Self-powered electro-tactile system for virtual tactile experiences. *Sci. Adv.* **7**, eabe2943 (2021).
37. Lee, S. *et al.* Nanomesh pressure sensor for monitoring finger manipulation without sensory interference. *Science* **370**, 966–970 (2020).
38. He, X. *et al.* Microstructured capacitive sensor with broad detection range and long-term stability for human activity detection. *npj Flex. Electron.* **5**, 1–9 (2021).
39. Shi, Y. *et al.* Eye tracking and eye expression decoding based on transparent, flexible and ultra-persistent electrostatic interface. *Nat. Commun.* **14**, 3315 (2023).
40. Chu, Y. *et al.* Human Pulse Diagnosis for Medical Assessments Using a Wearable Piezoelectret Sensing System. *Adv. Funct. Mater.* **28**, 1803413 (2018).
41. Park, D. Y. *et al.* Self-Powered Real-Time Arterial Pulse Monitoring Using Ultrathin Epidermal Piezoelectric Sensors. *Adv. Mater.* **29**, 1702308 (2017).
42. Yu, H. *et al.* Flexible temperature-pressure dual sensor based on 3D spiral thermoelectric Bi2Te3 films. *Nat. Commun.* **15**, 2521 (2024).
43. Huang, Y.-C. *et al.* Sensitive pressure sensors based on conductive microstructured air-gap gates and two-dimensional semiconductor transistors. *Nat. Electron.* **3**, 59–69 (2020).
44. Jang, J. *et al.* Mechanoluminescent, Air-Dielectric MoS2 Transistors as Active-Matrix Pressure Sensors for Wide Detection Ranges from Footsteps to Cellular Motions. *Nano Lett.* **20**, 66–74 (2020).

45. Lin, W. *et al.* Super-resolution wearable electrotactile rendering system. *Sci. Adv.* **8**, eabp8738 (2022).
46. Kajimoto, H. Electrotactile Display with Real-Time Impedance Feedback Using Pulse Width Modulation. *IEEE Trans. Haptics* **5**, 184–188 (2012).
47. Poletto, C. J. & Van Doren, C. L. A high voltage, constant current stimulator for electrocutaneous stimulation through small electrodes. *IEEE Trans. Biomed. Eng.* **46**, 929–936 (1999).
48. Root, S. E. *et al.* Ionotactile Stimulation: Nonvolatile Ionic Gels for Human–Machine Interfaces. *ACS Omega* **3**, 662–666 (2018).
