# Supplementary material for: Interference haptic stimulation and consistent quantitative tactility in transparent electrotactile screen with pressure-sensitive transistors
Source: Nat Commun. 2024 Aug 21;15:7147. doi: 10.1038/s41467-024-51593-2 (PMC11339070; doi:10.1038/s41467-024-51593-2)
Supplement: Supplementary file 3 — Description of Additional Supplementary Information [file 41467_2024_51593_MOESM3_ESM.pdf]

Description of Additional Supplementary Files

File Name: Supplementary Video 1
Description: Tactile information transmission through TPIEA.

File Name: Supplementary Video 2
Description: Display-integrated electrotactile sensation through TPIEA.

File Name: Supplementary Video 3
Description: Interference stimulation sensation perception test.
