# Supplementary material for: Interference haptic stimulation and consistent quantitative tactility in transparent electrotactile screen with pressure-sensitive transistors
Source: Nat Commun. 2024 Aug 21;15:7147. doi: 10.1038/s41467-024-51593-2 (PMC11339070; doi:10.1038/s41467-024-51593-2)
Supplement: Supplementary file 7 — Reporting Summary [file 41467_2024_51593_MOESM7_ESM.pdf]

nature portfolio

Corresponding author(s): Jang-Ung Park
Last updated by author(s): Aug 6, 2024

Reporting Summary

Nature Portfolio wishes to improve the reproducibility of the work that we publish. This form provides structure for consistency and transparency in reporting. For further information on Nature Portfolio policies, see our Editorial Policies and the Editorial Policy Checklist.

Statistics

For all statistical analyses, confirm that the following items are present in the figure legend, table legend, main text, or Methods section.

n/a	Confirmed	
☐	☒	The exact sample size (*n*) for each experimental group/condition, given as a discrete number and unit of measurement
☐	☒	A statement on whether measurements were taken from distinct samples or whether the same sample was measured repeatedly
☒	☐	The statistical test(s) used AND whether they are one- or two-sided<br>*Only common tests should be described solely by name; describe more complex techniques in the Methods section.*
☒	☐	A description of all covariates tested
☒	☐	A description of any assumptions or corrections, such as tests of normality and adjustment for multiple comparisons
☐	☒	A full description of the statistical parameters including central tendency (e.g. means) or other basic estimates (e.g. regression coefficient) AND variation (e.g. standard deviation) or associated estimates of uncertainty (e.g. confidence intervals)
☐	☒	For null hypothesis testing, the test statistic (e.g. *F*, *t*, *r*) with confidence intervals, effect sizes, degrees of freedom and *P* value noted<br>*Give P values as exact values whenever suitable.*
☒	☐	For Bayesian analysis, information on the choice of priors and Markov chain Monte Carlo settings
☒	☐	For hierarchical and complex designs, identification of the appropriate level for tests and full reporting of outcomes
☐	☒	Estimates of effect sizes (e.g. Cohen's *d*, Pearson's *r*), indicating how they were calculated

Our web collection on statistics for biologists contains articles on many of the points above.

Software and code

Policy information about availability of computer code

Data collection	Synapse (Tucker-Davis Technologies, Inc.)<br>VersaStudio<br>Keithley Model 2400 Series SourceMeter Software<br>Neuromaster G1 MEE-2000 (Nihon Kohden, Inc.)
Data analysis	Synapse (Tucker-Davis Technologies, Inc.)<br>Neuromaster G1 MEE-2000 (Nihon Kohden, Inc.)<br>ImageJ<br>Origin 2022b<br>Matblab R2020a<br>Excel

For manuscripts utilizing custom algorithms or software that are central to the research but not yet described in published literature, software must be made available to editors and reviewers. We strongly encourage code deposition in a community repository (e.g. GitHub). See the Nature Portfolio guidelines for submitting code & software for further information.

Data

Policy information about availability of data

All manuscripts must include a data availability statement. This statement should provide the following information, where applicable:

- Accession codes, unique identifiers, or web links for publicly available datasets
- A description of any restrictions on data availability
- For clinical datasets or third party data, please ensure that the statement adheres to our policy

The main data supporting the findings of this study are available within the paper and its Supplementary Information. Any additional requests for information can be directed to, and will be fulfilled by the corresponding authors. Source data are provided with this paper for reproducing all Figures in the manuscript and Supplementary Information.

Research involving human participants, their data, or biological material

Policy information about studies with human participants or human data. See also policy information about sex, gender (identity/presentation), and sexual orientation and race, ethnicity and racism.

Reporting on sex and gender	Sex and gender were not considered in the study design. The subjects in all experiments were individuals of both genders, between the ages of 22 and 40. Given that display and device usage are not restricted by gender or sex, we included both male and female subjects in our recruitment process.
Reporting on race, ethnicity, or other socially relevant groupings	There are no any relevant grouping experiments for race, ethnicity, or other socially.
Population characteristics	All subjects are over 18 years old. Total thirty healthy adult subjects (aged, 22-40 years; twenty female and ten male) were recruited, and randomly selected to each experiment. The number of people, gender, and age range for each experiment was recorded in detail in Methods.
Recruitment	The participants (healthy and adult) were recruited from campus of Yonsei University and neighboring communities through advertisement by posted notices and word of mouth. There were no self-selection biased or other biases.
Ethics oversight	The experiments with human subjects were performed in compliance with all the ethical regulations under a protocol that was approved by the Yonsei University Institutional Review Board (application no. 7001988-202303-HR-1809-02) via a haptic interface with no additional human-subject risk, following the provided study guidelines.

Note that full information on the approval of the study protocol must also be provided in the manuscript.

Field-specific reporting

Please select the one below that is the best fit for your research. If you are not sure, read the appropriate sections before making your selection.

[x] Life sciences [ ] Behavioural & social sciences [ ] Ecological, evolutionary & environmental sciences

For a reference copy of the document with all sections, see nature.com/documents/nr-reporting-summary-flat.pdf

Life sciences study design

All studies must disclose on these points even when the disclosure is negative.

Sample size	[Animal] In the mouse skin test, electrical stimulation set is applied with 0.015 A mm^-2 stimulation intensity, 10 Hz frequency, and 10 ms pulse width, followed by 1 minute of rest. 5 minutes of stimulation and rest are treated as 1 set. To check the short-term and long-term safety, the experiment was divided into four groups: Normal mouse, Normal mouse + 1 set of stimulation (6 minutes), Normal mouse + 5 sets of stimulation (30 minutes), and Normal mouse + 10 sets of stimulation (60 minutes). With a power of 0.8, a significance level of 0.05, and a large effect size, the number of samples required for one experiment is 14 mice. Each experiment was repeated three times. [Behavior Research Methods 39, 175–191 (2007).]<br>[Human] With subjects, we collected data at least 5 times and no more than 20 times for each cognitive test point to obtain significant cognitive test data, and repeated the experiment with at least 3 subjects and no more than 20 subjects for each experiment. The number of subjects was based on previous publication. [Sci. Adv.7,eabe2943(2021).] [Sci. Adv.8,eabp8738(2022).]
Data exclusions	No data were excluded from the analyses.
Replication	At least three biologically independent experiments were performed in each case of this study, and all experimental findings were reliably reproduced at least three times.
Randomization	All samples were allocated randomly into experminetal groups.
Blinding	As blocking visual information is an important issue in tactile perception experiments, all experiments were blinded: no surgical or chemical intervention was used, but rather a physical barrier (blindfold, cloth, etc.) to block the view.

Reporting for specific materials, systems and methods

We require information from authors about some types of materials, experimental systems and methods used in many studies. Here, indicate whether each material, system or method listed is relevant to your study. If you are not sure if a list item applies to your research, read the appropriate section before selecting a response.

Materials & experimental systems

n/a	Involved in the study
☒	☐ Antibodies
☐	☒ Eukaryotic cell lines
☒	☐ Palaeontology and archaeology
☐	☒ Animals and other organisms
☒	☐ Clinical data
☒	☐ Dual use research of concern
☒	☐ Plants

Methods

n/a	Involved in the study
☒	☐ ChIP-seq
☒	☐ Flow cytometry
☒	☐ MRI-based neuroimaging

Eukaryotic cell lines

Policy information about cell lines and Sex and Gender in Research

Cell line source(s)	SH-SY5Y was purchased from Korean Cell Line Bank (KCLB 22266, Lot# 51972)
Authentication	Cell line was authenticated by company that we ordered from. Morphology check by microscope periodically. Cell morphology was similar to published pictures.
Mycoplasma contamination	The cell was used under mycoplasma-free condition
Commonly misidentified lines (See ICLAC register)	No commonly misidentified cell lines were used in this study.

Animals and other research organisms

Policy information about studies involving animals; ARRIVE guidelines recommended for reporting animal research, and Sex and Gender in Research

Laboratory animals	C57BL/6 mice (male, 6 weeks, 20~25g, total 14 mice) were purchased from ORIENT BIO Inc(Korea, Repulic of). The mice were raised in a specific pathogen free (SPF) environment with an ambient temperature of 23℃, a humidity of 50%, and a 12 h dark/light cycle.
Wild animals	This study did not involve wild animals
Reporting on sex	Sex was not considered in the study.
Field-collected samples	This study did not involve samples collected from the field
Ethics oversight	The experiments with mouse was performed in compliance with all the ethical regulations under a protocol that was approved by the Institutional Animal Care and Use Committee of Yonsei University (application no. IACUC-A-202405-1851-01)

Note that full information on the approval of the study protocol must also be provided in the manuscript.

Plants

Seed stocks	The study did not involve plant samples
Novel plant genotypes	The study did not involve plant samples
Authentication	The study did not involve plant samples
